# Principles of weakly ordered domains in intermetallics: the cooperative effects of atomic packing and electronics in Fe_2_Al_5_

**DOI:** 10.1107/S2053273318017461

**Published:** 2019-02-21

**Authors:** Anastasiya I. Vinokur, Katerina P. Hilleke, Daniel C. Fredrickson

**Affiliations:** a Department of Chemistry, University of Wisconsin-Madison, 1101 University Avenue, Madison, WI 53706, USA

**Keywords:** intermetallic phases, channel structures, chemical bonding theory, disorder, incommensurability

## Abstract

Many complex intermetallic structures feature a curious juxtaposition of domains with strict 3D periodicity and regions of much weaker order or incommensurability. This article explores the basic principles leading to such arrangements through an investigation of the weakly ordered channels of Fe_2_Al_5_. It starts by experimentally confirming the earlier crystallographic model of the high-temperature form, in which nearly continuous columns of electron density corresponding to disordered Al atoms emerge. Then electronic structure calculations on ordered models are used to determine the factors leading to the formation of these columns. These calculations reveal electronic pseudogaps near 16 electrons/Fe atom, an electron concentration close to the Al-rich side of the phase’s homogeneity range. Through a reversed approximation Molecular Orbital (raMO) analysis, these pseudogaps are correlated with the filling of 18-electron configurations on the Fe atoms with the support of isolobal σ Fe–Fe bonds. The resulting preference for 16 electrons/Fe requires a fractional number of Al atoms in the Fe_2_Al_5_ unit cell. Density functional theory–chemical pressure (DFT-CP) analysis is then applied to investigate how this nonstoichiometry is accommodated. The CP schemes reveal strong quadrupolar distributions on the Al atoms of the channels, suggestive of soft atomic motions along the undulating electron density observed in the Fourier map that allow the Al positions to shift easily in response to compositional changes. Such a combination of preferred electron counts tied to stoichiometry and continuous paths of CP quadrupoles could provide predictive indicators for the emergence of channels of disordered or incommensurately spaced atoms in intermetallic structures.

## Abstract 2

The Fe_2_Al_5_ structure is remarkable among intermetallic phases for its channels of weakly ordered Al atoms. This article traces the origins of these channels to the cooperative effects of soft atomic motions dictated by chemical pressure quadrupoles and preferred electron concentrations.

## 1. Introduction   

The structural chemistry of intermetallic phases is rich in phenomena that go beyond simple notions of translational symmetry in 3D space, such as disorder, incommensurability and quasicrystalline arrangements. In each of these scenarios, weakly specified atomic positions are often distributed within frameworks of rigidly positioned atoms with a variety of morphologies. In some cases, such as the disordered tetrahedra at the centers of the icosahedral building units of Tsai-type quasicrystals and their approximants (Gómez & Lidin, 2003 ▸; Piao *et al.*, 2006 ▸; Takakura *et al.*, 2007 ▸), the less organized regions can be isolated to small pockets of the structure. Disordered or incommensurately spaced atoms can also run along features of higher dimensionality: 1D columns (Mählpfordt, 1997 ▸; Rohrer *et al.*, 2001 ▸; Piao & Lidin, 2007 ▸; Kanno *et al.*, 2017 ▸) or 2D planes (Latturner & Kanatzidis, 2002 ▸; Gray *et al.*, 2008 ▸; Kilduff & Fredrickson, 2016 ▸). In the most extreme examples, as in the classic β-Mg_2_Al_3_ structure (Samson, 1965 ▸; Feuerbacher *et al.*, 2007 ▸), the scrambled atoms can form 3D continuous sublattices that permeate the whole crystal. Such a duality of strict and loose order within the same structure offers immense opportunities for material properties, *e.g.* for the realization of the phonon-glass electron crystal concept (Nolas *et al.*, 1999 ▸), or the creation of well defined paths for atomic diffusion (Mehrer, 1996 ▸). However, the absence of periodic order in these situations makes their design from first-principles calculations challenging. In this article, we will work towards deriving chemical principles for the emergence of such features in intermetallics, using the channels of weakly ordered atoms in the Fe_2_Al_5_ structure as a model system.

**Figure 1:**
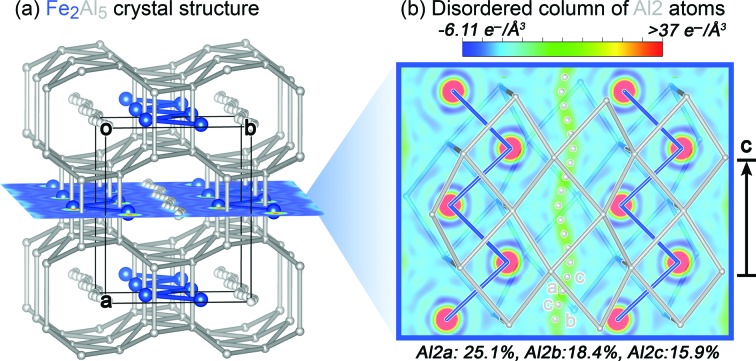
The high-temperature disordered form of Fe_2_Al_5_. (*a*) View of the crystal structure approximately down the *c* axis. (*b*) The channel of electron density along the *c* axis modeled in our structural solution with three partially occupied Al sites: Al2a–c.

As with many other non-trivial intermetallic compounds, the structure of Fe_2_Al_5_ (a phase whose homogeneity range extends from approximately Fe_2_Al_4.67_ to Fe_2_Al_5.4_) has gradually emerged over the course of decades in successive investigations. After an initial report of an FeAl_2_ phase in 1933 (Osawa, 1933 ▸), the stoichiometry of the phase was revised in 1953 to Fe_2_Al_5_, and the compound was described in passing as being structurally similar to the hexagonal phase Co_2_Al_5_ (Schubert *et al.*, 1953 ▸). Subsequent investigations revealed that, on the contrary, it adopts its own structure type with little relation to that of Co_2_Al_5_ (Van der Kraan & Buschow, 1986 ▸; Ellner & Mayer, 1992 ▸). In 1994, a crystal structure determination revealed nearly continuous columns of electron density passing through the structure (Fig. 1 ▸), which were modeled as chains of tightly spaced, fractionally occupied Al atoms (Burkhardt *et al.*, 1994 ▸). This extensive positional disorder inspired numerous investigations into the phase’s properties and stability. Fe_2_Al_5_ was discovered to behave as a dilute magnet (Chi *et al.*, 2010 ▸; Jagličić *et al.*, 2011 ▸), while phonon band structure calculations revealed fluidity along the disordered channels (Mihalkovič & Widom, 2012 ▸) in accord with the heat capacity measurements of Chi *et al.* (2010 ▸). Such soft motions are suggestive of low thermal conductivity, highlighting potential for thermoelectric applications that was recently realized experimentally (Tobita *et al.*, 2016 ▸). Finally, in the latest structural studies, low-temperature polymorphs exhibiting superstructure formation or incommensurate order were characterized with selected-area electron diffraction and powder X-ray diffraction (Becker *et al.*, 2017 ▸; Okamoto *et al.*, 2017 ▸; Becker & Leineweber, 2018 ▸).

As the behavior of the Al atoms in this column and the consequent potential for physical properties have emerged with greater clarity, a number of questions of structural chemistry remain unresolved. What drives the formation of these weakly ordered channels? How could similar situations be staged in other intermetallic compounds? Motivated by these questions, we will investigate the electronic structure of Fe_2_Al_5_, correlating its structural features to the 18 − *n* bonding scheme (Yannello & Fredrickson, 2014 ▸, 2015 ▸) and to chemical pressure (CP) distributions (Fredrickson, 2012 ▸; Berns *et al.*, 2014 ▸). Using the reversed approximation Mol­ecular Orbital (raMO) analysis (Yannello *et al.*, 2014 ▸), the Al nonstoichiometry will be linked to the preferred electron count of the Fe bonding network. The localization of the nonstoichiometry to the weakly ordered channels will then be connected to the presence of CP quadrupoles on the corresponding Al atoms, a feature associated with soft atomic motions (Engelkemier & Fredrickson, 2016 ▸). Fe_2_Al_5_ will thus exemplify complex structures in which disordered regions arise from cooperation between electronic and atomic packing effects, where a feature driven by electronic effects is guided by CP, a theme we anticipate to play a broad role in intermetallic chemistry.

## 2. Technical procedures   

### 2.1. Experimental procedures   

To confirm its structural features, the high-temperature form of Fe_2_Al_5_ was synthesized by first grinding together powders of the pure elements (Fe powder, 99.9%, Strem; Al powder −100+325 mesh, 99.97%, Alfa Aesar) in a ratio of 1:2.7 with an agate mortar and pestle in an Ar-filled glovebox. The mixtures were then pressed into pellets and arc-melted under Ar two times for 10 s each (to maximize homogeneity while minimizing Al loss by evaporation). The pellets were sealed in evacuated fused silica ampoules and annealed first at 873 K for 4 d, then at 673 K for 6 d, and finally quenched in ice water. All syntheses resulted in gray pellets with a metallic sheen that showed no visible signs of air sensitivity even after weeks in air. As described in the supporting information, the samples were characterized with single-crystal and powder X-ray diffraction, energy-dispersive X-ray spectroscopy (EDS), and wavelength-dispersive X-ray spectroscopy (WDS).

### 2.2. Ordered structure models   

For the theoretical analysis of the driving forces shaping the disordered regions within Fe_2_Al_5_, several ordering patterns were examined involving different placements of Al atoms over the Al2a, Al2b and Al2c sites within the two channels in the conventional cell. Five models in total were considered (designated as Models 1–5) following the occupation patterns given in Table S14 in the supporting information. For example, Model 1 was built from a 1 × 1 × 2 supercell, with only every fifth atom along the Al2 Al columns being kept, then deleting one additional Al atom from one of the columns, generating the appropriate Fe_2_Al_5.25_ stoichiometry (15.875 electrons/Fe) and a space-group symmetry of *Pm*11 (with the non-standard setting being used to maintain the axis labels of the original cell). For Model 2, Al atoms were placed at every other Al2a site (space group *C*2/*m*11, also set in a 1 × 1 × 2 supercell in the calculations preliminary to the raMO analysis, for consistency), yielding a stoichiometry of Fe_2_Al_5_ (15.5 electrons/Fe). In Model 3, meanwhile, every other Al2b site is occupied by an Al atom (stoichiometry: Fe_2_Al_5_, space group: *Cm*2*m*). Models 4 and 5 are variations on Models 1 and 2, respectively, in which the contents of the two weakly ordered columns in the conventional cell are shifted relative to each other, with the symmetries *Pm*11 and *Pmnn* (though, curiously, the geometrical optimization of Model 4 converged on a geometry nearly indistinguishable from Model 1). Models 1 and 2 were used for the raMO analysis, with the similar electronic density of states (DOS) features of the remaining models (Fig. S7 in the supporting information) suggesting that analogous bonding schemes apply. Density functional theory–chemical pressure (DFT-CP) analysis was carried out on all of the unique structures (Models 1–3 and 5). The stoichiometries for all models lie within the experimental phase width of the phase, where the electron concentration ranges from 15.0 to 16.1 electrons/Fe (Li *et al.*, 2016 ▸).

### 2.3. Reversed approximation Molecular Orbital analysis   

The raMO analysis was employed to analyze the role of electron count in the stability of Fe_2_Al_5_. The DFT electronic structures, under the generalized gradient approximation (GGA), of Models 1 and 2 were calculated with the *Vienna Ab Initio Simulation Package* (*VASP*) (Kresse & Furthmüller, 1996*a* ▸,*b* ▸) in the high-precision mode, using the projector augmented wave (PAW) potentials provided with the program (Blöchl, 1994 ▸; Kresse & Joubert, 1999 ▸), 3 × 3 × 3 Γ-centered *k*-point grids and an energy cutoff of 334.9 eV. Both structures were geometrically optimized with a two-step procedure, with the atomic coordinates first being relaxed within a fixed unit cell, followed by the release of all structural parameters. Single-point calculations were then performed to obtain band energies and DOS distributions.

The resulting GGA-DFT output was then used to refine parameters for simple Hückel models with the program *eHtuner* (Stacey & Fredrickson, 2012 ▸). From the finalized Hückel parameters, Hamiltonian matrices were calculated with *YAeHMOP* (Landrum, G. A. & Glassey, W. V.; *YAeHMOP: Yet Another extended Hückel Molecular Orbital Package*, freely available at http://yaehmop.sourceforge.net/) for the Γ points of 3 × 3 × 3 supercells, which were directly input into our in-house MATLAB programs *Figuretool2* and *makeraMO* for the raMO analyses (Yannello *et al.*, 2014 ▸). Further details, including atomic coordinates for the models, tables of DFT-calibrated Hückel parameters and comparisons of the GGA-DFT and Hückel DOS curves, are provided in the supporting information.

### 2.4. DFT-CP analysis   

DFT-CP analyses were performed on Models 1–3 and 5. Prior to the DFT-CP calculations, geometry optimizations were performed with DFT under the local density approximation (LDA) with the *ABINIT* program (Gonze *et al.*, 2002 ▸, 2009 ▸) using Hartwigsen–Goedecker–Hutter norm-conserving pseudopotentials (Hartwigsen *et al.*, 1998 ▸) for Models 2, 3 and 5. Because of its larger cell size, the geometry optimizations for Model 1 were performed using LDA-DFT in *VASP* with ultrasoft pseudopotentials (Vanderbilt, 1990 ▸). Single-point calculations were then carried out in *ABINIT* on these optimized geometries at the equilibrium volume as well as at slightly expanded and contracted volumes, yielding the kinetic energy densities, electron densities and local components of the Kohn–Sham potentials used in the CP analyses. Further details, including energy cutoffs and *k*-point grids, may be found in the supporting information.

CP maps were produced from the *ABINIT* output using the *CPmap* module of *CPpackage2*, including core unwarping and mapping of the nonlocal pseudopotential energies (Berns *et al.*, 2014 ▸; Hilleke & Fredrickson, 2018 ▸). These maps were divided among contact volumes between pairs of atoms using the binary Hirshfeld-inspired scheme in the *CPintegrate* module (Berns *et al.*, 2014 ▸), averaging the CP values within the contact volumes to obtain interatomic pressures. These pressures were next projected onto real spherical harmonics (*l* ≤ 4) centered on the atomic positions, and the resulting CP schemes were visualized using *Figuretool2*. The free ion electron densities used in the core unwarping and contact volume construction procedures were created with the *Atomic Pseudopotentials Engine* (*APE*) (Oliveira & Nogueira, 2008 ▸), using fractions of the charges obtained from a Bader charge analysis of the PAW-GGA electronic structures with the Bader program (Bader, 1990 ▸; Henkelman *et al.*, 2006 ▸; Tang *et al.*, 2009 ▸) or using the Hirshfeld charges (Hirshfeld, 1977 ▸) output by *CPpackage2*.

### 2.5. Phonon band structure calculations   

The phonon band structure of Model 2 (with every other Al2a site occupied) used for the CP analysis was calculated with the *ABINIT* program using the linear response method (Giannozzi *et al.*, 1991 ▸). A series of calculations were carried out, beginning with the production of a wavefunction file using a Γ-centered *k*-point grid. Subsequent non-self-consistent calculations were performed at all *q*-points corresponding to the *k*-points used in the reference calculation to obtain linear responses for displacements of individual atoms along each Cartesian axis. The interatomic force constants were determined from these linear responses using the *ABINIT* utilities *mrgddb* and *anaddb*, while the resulting phonon modes, band structures and DOS curves were plotted with *Figuretool2*.

## 3. Results and discussion   

### 3.1. Structural analysis of the high-temperature Fe_2_Al_5_ phase   

With an interest in the weakly ordered channels of Fe_2_Al_5_, we synthesized samples of its high-temperature form for structural analysis. Single crystals were picked from the crushed reaction mixture for X-ray diffraction experiments. The diffraction data collected at room temperature were indexed with an orthorhombic unit cell with *a* = 7.5660 (3), *b* = 6.4117 (2) and *c* = 4.22350 (18) Å, in close agreement with the previously reported cell (Burkhardt *et al.*, 1994 ▸). No superstructure or satellite reflections were observed, all of the observed systematic absences were consistent with the *Cmcm* space group assigned earlier, and the structure solution shows features in accord with those Burkhardt *et al.* chronicled.

**Figure 2:**
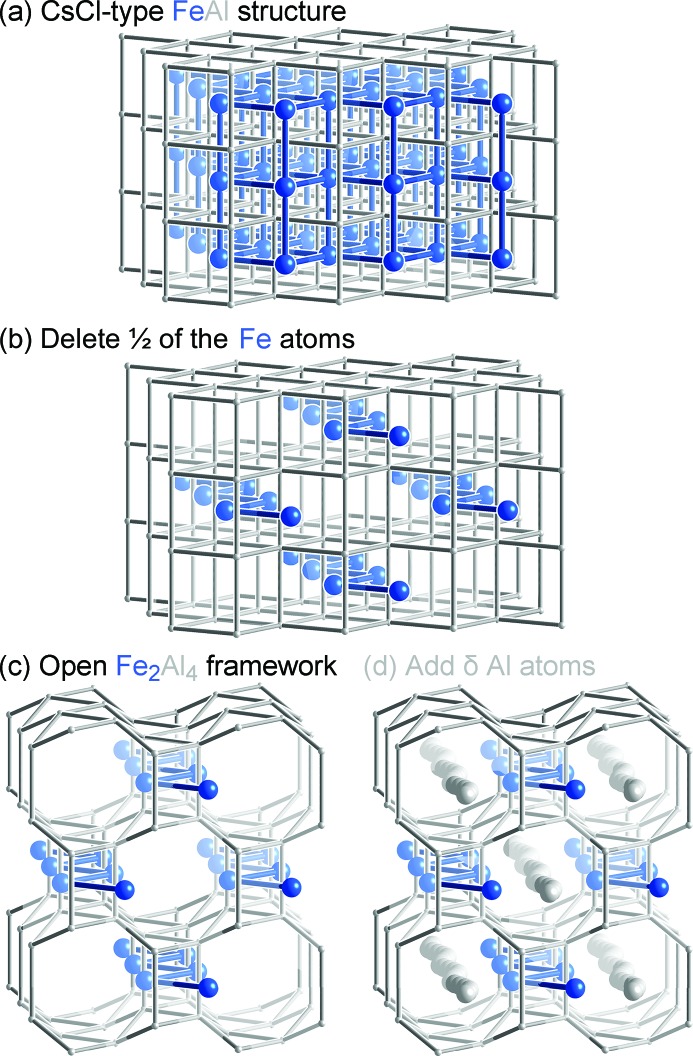
Derivation of the Fe_2_Al_5_ structure. (*a*) The CsCl-type structure of FeAl. (*b*) Deletion of half of the Fe atoms of FeAl such that the remaining ones form zigzag chains. (*c*) Opening the resulting Fe_2_Al_4_ framework creates channels ready for the inclusion of additional atoms. (*d*) The addition of δ Al atoms into the channels yields a structure with the composition Fe_2_Al_4+δ_.

A simple way to visualize this structure that will be helpful for our theoretical analysis is through its relationship to the CsCl-type structure of FeAl (Fig. 2 ▸ *a*). To derive the Fe_2_Al_5_ structure, we first delete half of the Fe atoms in FeAl (Fig. 2 ▸ *b*) to leave behind Fe zigzag chains, creating a Fe_2_Al_4_ framework. We next open the framework around the new void spaces (Fig. 2 ▸ *c*) to form octagonal channels. Finally, the structure is completed by adding Al atoms in a disordered chain into the channel, yielding a composition of Fe_2_Al_4+δ_ [δ ≃ 0.67 to 1.4 (Fig. 2 ▸ *d*)]. In effect, the structure is derived from FeAl by replacing half the Fe atoms with continuous columns of possible Al positions.

The Fourier map for the Al atoms in this channel shows an uninterrupted, sinusoidal column of electron density (Fig. 1 ▸). Modeling this region atomistically is somewhat arbitrary given the absence of features corresponding to discrete positions. We found it convenient to model the domain with the three partially occupied Al positions shown in Fig. 1 ▸(*b*), where two of the sites (Al2a and Al2b) correspond to maxima in the density and the third (Al2c) serves to bridge them. Curiously, the Al content of the column consistently refined to a formula near Fe_2_Al_5.5_ for various models, compared with the Fe_2_Al_5.24_ determined from our WDS measurements. Attempts to constrain the Al occupancies to match the WDS result, however, led some of the Al atomic displacement parameters to become nonpositive definite. Such overestimation of Al content in the crystallographic model was also reported by Burkhardt *et al.*, which they attributed to an incomplete absorption correction or some Fe mixing on the disordered Al sites. The presence of a small amount of Fe in this disordered region would easily explain the discrepancy between the WDS and single-crystal compositions, and was included in the models of Becker & Leineweber (2018 ▸). However, for our present purposes, we will focus on the role Al atoms play in these channels, neglecting the potential presence of a minor number of Fe atoms.

**Figure 3:**
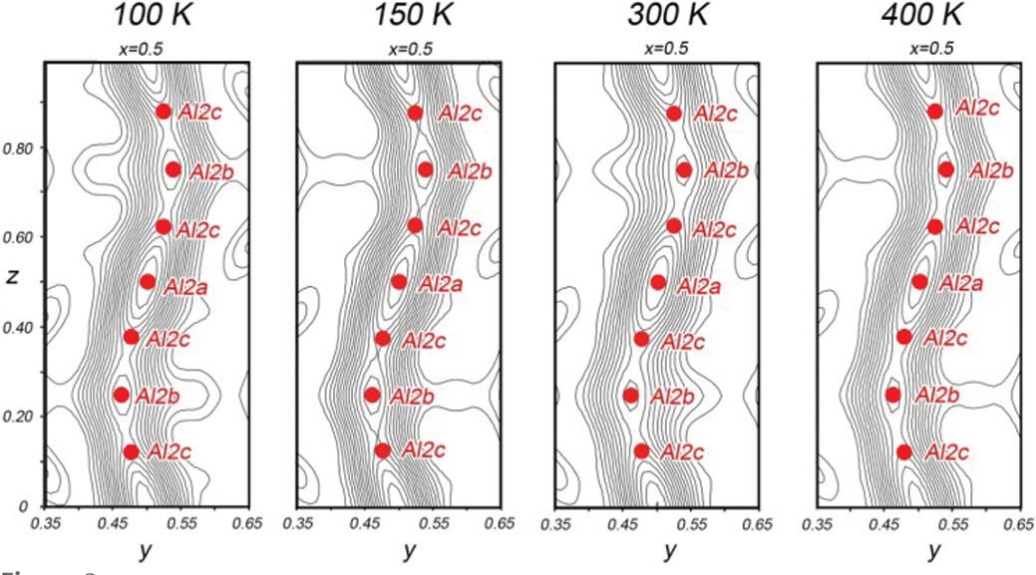
Evolution of the X-ray diffraction pattern and Al2a, Al2b and Al2c channel electron density as a function of temperature. The cross sections of the Fourier electron density through the Al2a, Al2b and Al2c channels obtained for data sets collected at 100, 150, 300 and 400 K.

While neither superstructure nor satellite reflections were observed at room temperature, we were curious whether ordering transitions might be observable under different conditions. We thus carried out single-crystal X-ray diffraction experiments at 100, 150 and 400 K. In each case, no additional reflections appeared (see the supporting information), and solving the crystal structures at the three temperatures showed the same continuous column of electron density, which was still well modeled with a triad of partially occupied Al positions (Fig. 3 ▸), and showed only minimal shifts in the atom positions. For the relatively Al-rich composition of the crystal, it thus appears that the kinetics for transforming to the ordered polymorphs of Fe_2_Al_5_ are slow at these temperatures relative to the time-scale of the single-crystal data collection. Together, these observations highlight the weakness of the driving forces for positional order along the structure’s channels.

### 3.2. Electron counting and Al nonstoichiometry   

In our structural analysis of the last section, as in previous studies, the most striking characteristic of Fe_2_Al_5_ is its channels of weakly ordered Al atoms. We now turn to exploring the role that these features play in the stability of Fe_2_Al_5_. The composition of this compound places it within the domain of a broad electron counting rule for transition metal–main group (T–E) intermetallic phases, the 18 − *n* rule (Yannello & Fredrickson, 2014 ▸, 2015 ▸), which can be considered as a limiting case of the more general 5*t* + 4*c* − *b* scheme of Kitahara *et al.* (2015 ▸, 2017 ▸). In these compounds, the T atoms strive for filled 18-electron configurations, in which an electron pair is associated with each of their valence *s*, *p* and *d* atomic orbitals. To achieve these closed shells, each T atom requires 18 − *n* electrons, where *n* is the number of electron pairs the T atom shares covalently with its T-atom neighbors through multicenter E-bridged functions we refer to as isolobal T–T bonds (Yannello & Fredrickson, 2014 ▸, 2015 ▸). Adherence to this rule is correlated with the presence of a bandgap or pseudogap in the DOS distributions at the Fermi energy (*E*_F_), common indicators of electronic stability.

One advantage of the 18 − *n* rule is that it can be simply applied to compounds where disorder occurs in the main group sublattice, as only the total electron concentration of the phase and the T–T connectivity must be definitively known. According to the Fe–Al phase diagram (Li *et al.*, 2016 ▸), for temperatures below *circa* 1273 K, the Fe_2_Al_5_ homogeneity range stretches from 70.0 to 72.9 at.% Al, leading to a range of valence electron counts of 15.0 to 16.1 electrons/Fe atom. As the upper end of this range is approximately two electrons/Fe atom short of 18, we expect the Fe atoms should each take part in two Fe–Fe isolobal bonds, consistent with their condensation into zigzag chains in Fe_2_Al_5_.

**Figure 4:**
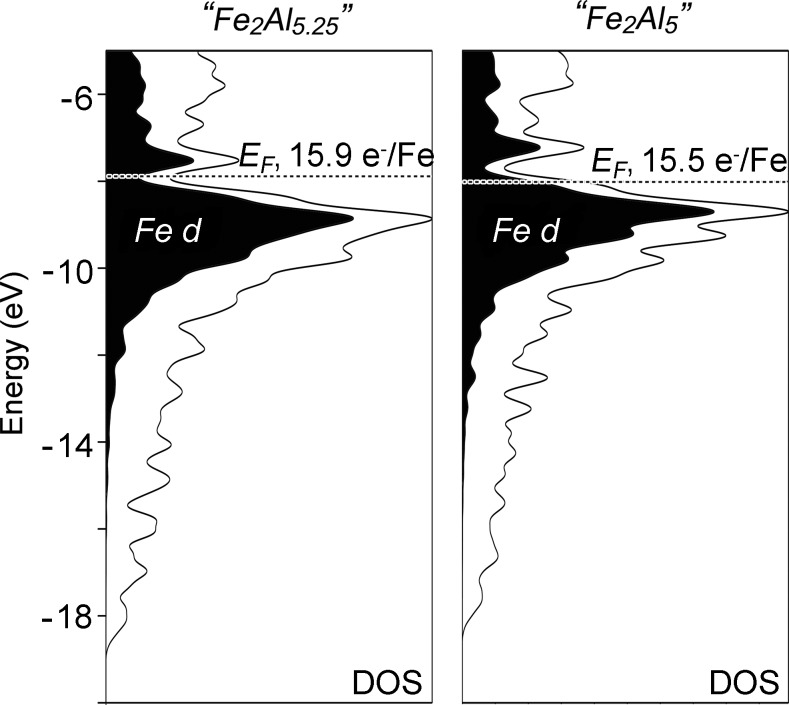
DFT electronic DOS distributions for two ordered models of Fe_2_Al_5_ (left: Model 1, right: Model 2). Gaussian broadening has been applied here (and in other DOS curves in this work) to bring out their more general features.

Testing this picture with theoretical calculations, however, requires periodic, ordered models of the structure. As described in Section 2.2, we constructed two such GGA-DFT-optimized models for detailed bonding analysis with stoichiometries of Fe_2_Al_5.25_ (Model 1) and Fe_2_Al_5_ (Model 2). The resulting electronic DOS distributions (Fig. 4 ▸) show strong similarities despite the differences in the placement and loading of the Al2 atoms within the channels. For each model, the *E*_F_ falls close to a narrow pseudogap centered near −8 eV (hinting at nearly optimized bonding), lying right in the center of the DOS minimum at 15.86 electrons/Fe (Fe_2_Al_5.25_), and dropping to the lower shoulder at 15.5 electrons/Fe (Fe_2_Al_5_). The states bracketing the pseudogap in each case are largely derived from the Fe *d* orbitals (whose contributions are shaded in black), while the lowest energy levels (between −12 and −19 eV) are dominated by a nearly free electron-like distribution rich in Al *sp* character.

The presence of a pseudogap near 16 electrons/Fe atom is highly suggestive of the compound following the 18 − *n* scheme. To make this connection more direct, we turn to the raMO analysis (Yannello *et al.*, 2014 ▸). In the raMO approach, the occupied crystal orbitals of a system serve as a basis set for the reconstruction of localized target functions which are hypothesized to play a key role in the local bonding of the compound, for an 18-electron configuration usually the T atom’s nine *s*, *p* and *d* valence orbitals. The raMO analysis then creates Wannier-like functions that represent the best possible orthogonal approximations to these target functions from the occupied orbitals, with the deviations from the original targets reflecting their bonding context and occupation.

**Figure 5:**
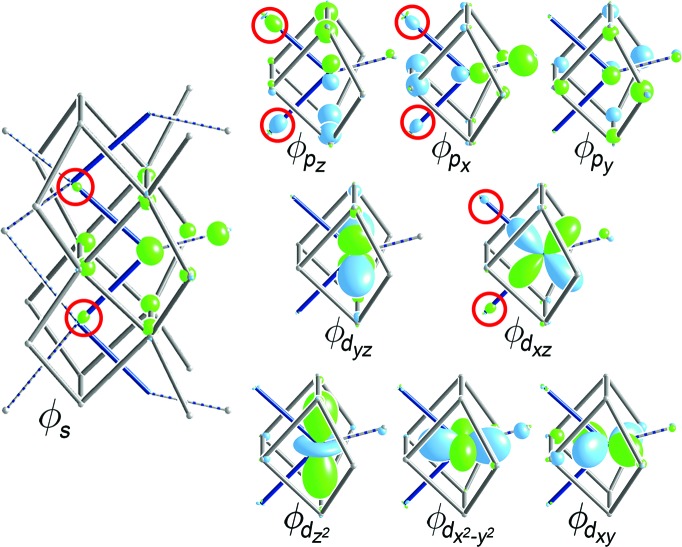
raMO reconstructions of the 4*s*, 4*p* and 3*d* valence orbitals for an Fe atom in the Fe_2_Al_5_ phase (Model 1, composition: Fe_2_Al_5.25_). Substantial contributions from neighboring Fe sites are circled in red. Additional raMO results are provided in the supporting information.

In Fig. 5 ▸, we show the raMO reconstructions of the nine valence *s*, *p* and *d* orbitals for an Fe atom in Model 1, generated using a DFT-calibrated Hückel model. At the center of each raMO, the original target atomic orbital can be seen serving as the core of a function that spreads to varying degrees around the first coordination environment through bonding interactions. This localization of the function to the Fe atom and its immediate surroundings is consistent with the notion that each of these states is occupied by an electron pair, providing the Fe atom with a filled 18-electron configuration.

**Figure 6:**
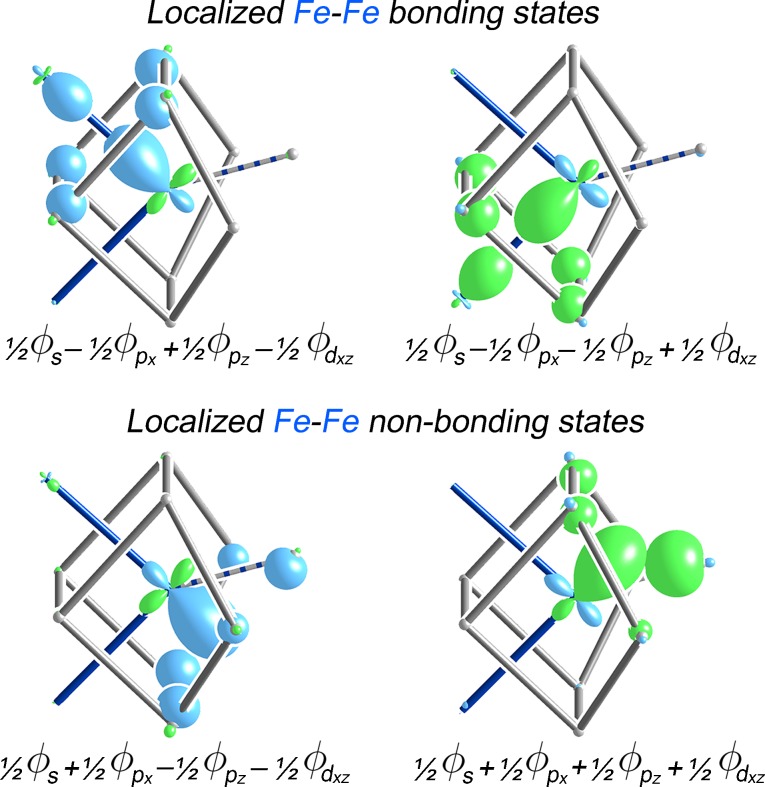
*sp* ^2^ *d* hybrids constructed from the raMOs in Fig. 5 ▸, revealing two isolobal Fe–Fe bonds, as well as two Fe–Fe nonbonding functions.

However, this configuration cannot be completely assigned to the central Fe atom independently: four of the raMOs (those based on the 4*s*, 4*p_x_*, 4*p_z_* and 3*d_xz_* orbitals) exhibit bonding contributions from the neighboring Fe atoms in the zigzag chain, indicative of electron sharing between the Fe atoms (Fig. 5 ▸, red circles). The four atomic orbitals at the centers of these raMOs in fact form a set appropriate to the creation of *sp* ^2^ *d* hybrid orbitals pointing to the corners of a square, which would closely match the ∼90° Fe–Fe–Fe angles in the chains. Taking such linear combinations of these four raMOs produces two bonding functions localized along individual Fe–Fe contacts representing Fe–Fe isolobal σ bonds (Fig. 6 ▸, top). The remaining two states generated by these linear combinations have the lobes pointing outwards from the Fe chain towards the neighboring Al2 column (Fig. 6 ▸, bottom). These would be considered non-bonding Fe–Fe interactions, resembling Fe lone pairs that coordinate to the surrounding Al atoms. The remaining five raMOs not involved in this hybridization are also classified as Fe–Fe nonbonding functions.

The two Fe–Fe isolobal bonds emerging from this raMO analysis concur with the hypothesis that Fe_2_Al_5_ follows the 18 − *n* bonding scheme with *n* = 2, such that the electronic structure is optimized at 16 electrons/Fe atom. As such, the reasons for the nonstoichiometry in the Al sublattice become apparent. A perfect 16 electron count requires a stoichiometry of Fe_2_Al_5.33_, which corresponds to a desired Al content of 10.667 atoms (a non-integer number) for the four Fe atoms within the Fe_2_Al_5_ unit cell. The Al-rich side of the experimentally observed homogeneity range, with electron concentrations up to 16.1, coincides closely with filling the band structure up to the top of the pseudogap. Adding more Al atoms into the system would then be expected to populate Fe–Fe σ* antibonding states, destabilizing the structure. The phase appears to be more flexible with lower electron counts, with electron counts as low as 15.0 in Al-poor compositions apparently being accessible. This greater flexibility towards electron-poor configurations over electron-rich ones is commonly observed in structures governed by the 18 − *n* bonding scheme (Yannello & Fredrickson, 2015 ▸), as the lower electron counts can open opportunities for isolobal π interactions.

### 3.3. Chemical pressure quadrupoles along the disordered channel   

Thus far, we have traced the Al nonstoichiometry in Fe_2_Al_5_ to the phase’s adherence to the 18 − *n* bonding scheme. This picture, however, focuses almost entirely on the Fe atoms and their placement relative to each other; the Al atoms merely serve to provide electrons and support functions whose symmetry properties are templated by the Fe atomic orbitals. As such, we gain little information about how the Al nonstoichiometry should be distributed through the unit cell and, in particular, why it becomes concentrated into weakly ordered columns running through the structure. In this section, we will see how the DFT-CP analysis accounts for these observations and suggests a general framework for identifying portions of a structure susceptible to weak positional order.

In the CP analysis, the local strains in the atomic packing of a compound are visualized by decomposing its macroscopic pressure, as obtained from DFT, into a spatially resolved map of regions of negative and positive pressure calling for contraction and expansion of the structure, respectively. These local pressures are analyzed in terms of inter­atomic inter­actions by dividing space into volumes corresponding to pairs of atoms and averaging the pressures within to obtain inter­atomic pressures, which are then projected onto spherical harmonics. The pressures experienced by each atom are finally displayed with radial plots, where black lobes represent negative CP (desire for contraction), white ones denote positive CP (desire for expansion), and the distance from the nucleus to the surface is proportional to the sum of the CP contributions along each direction.

**Figure 7:**
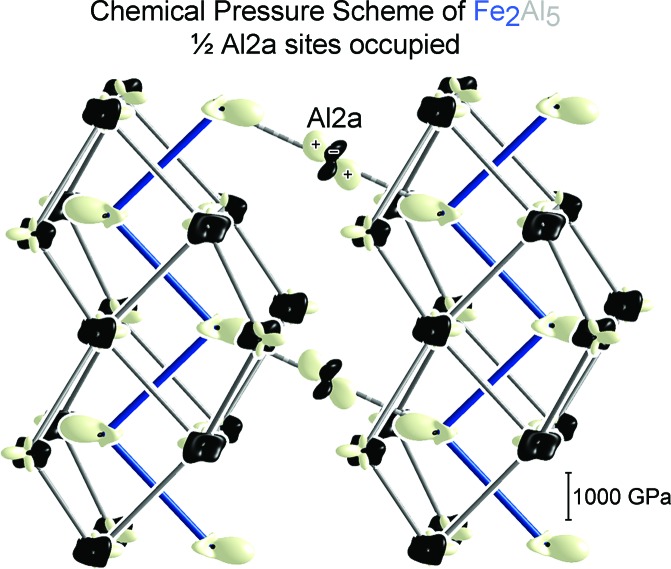
DFT-CP scheme for an ordered model of Fe_2_Al_5_ in which Al atoms are placed at one half of the Al2a sites (Model 2, see Fig. 1 ▸). The conventions of the CP plots are described in the text.

In Fig. 7 ▸, we show as an example the CP scheme for one model of Fe_2_Al_5_ in which Al atoms are placed on one half of the Al2a sites (Model 2). The Fe–Al interactions are marked by prominent lobes of positive CP calling for expansion of the structure – strongest between Fe and the Al2a atoms in the channel but also present between Fe and the Al atoms in the Fe_2_Al_4_ host lattice. These calls, however, are resisted by negative pressures on the Al atoms that, while less focused, lie along Al–Al interactions. The major tension in the atomic packing of Fe_2_Al_5_ thus seems to stem from the overly compressed Fe–Al interactions whose release is stymied by the already too-stretched Al sublattice.

One of the most striking aspects of this CP scheme is the distribution of pressures around the Al2a sites. These atoms are placed directly between two Fe neighbors on opposite sides of the channel. This results in the Al atom experiencing strong positive CPs along its close Al–Fe contacts, pinning it to the center of the channel. Meanwhile, negative CP features on the Al atoms are perpendicular to the positive CP and slanted along the local direction of the channel, reflecting the sparse­ness of the Al channel population. Such a quadrupolar CP distribution is frequently associated with soft atomic motions, as shifts along the direction of negative pressure would serve to shorten some overly long contacts while relieving positive CPs in the perpendicular direction.

**Figure 8:**
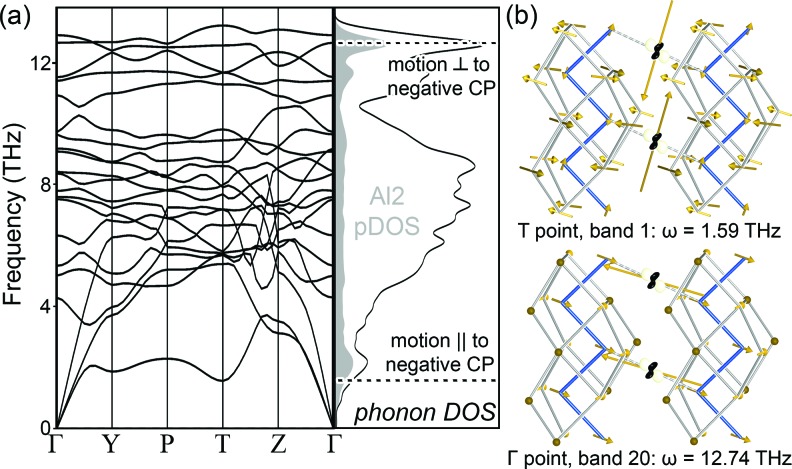
Connection between CP quadrupoles and phonon modes. (*a*) Phonon band structure and DOS for the model Fe_2_Al_5_ compound with one half of the Al2a sites occupied (Model 2). The projected phonon DOS for the Al2a site is highlighted in gray. (*b*) Selected phonon modes from the peaks in the Al2a projected DOS show anisotropy that aligns with the CP quadrupole of the Al2a site (overlaid).

To explore this possibility of soft motions, we carried out calculations on the phonon band structure of this ordered model of Fe_2_Al_5_ (Fig. 8 ▸ *a*). As Al is significantly lighter than Fe, we would normally expect Al-based motions to dominate the high-frequency modes, with the Fe atoms participating in the low-frequency vibrations. It is remarkable, then, that the lowest peak in the DOS stems chiefly from the motions of the Al2 sites (shaded in gray) in a single low-frequency band.

To examine the character of this band, we inspect its phonon mode at the *q*-point *T* (½ ½ ½) in the top panel of Fig. 8 ▸(*b*), where the atomic motions are represented with arrows. The largest amplitudes appear on the Al2 atoms along directions of negative CP, corresponding to a motion along the undulating Al2 disordered column in the experimental structure. A second peak with substantial Al2 contributions can be found in the phonon DOS near the top of the band structure at *circa* 12.7 THz. In stark contrast to the character of the low-frequency mode, these modes correspond to motions of the Al2 atom directly along the positive Fe–Al CP [Fig. 8 ▸(*b*), bottom]. The anisotropy of the CP distribution around the Al2 atoms, with negative CP along the channel and positive CP towards the Fe-lined channel walls, is therefore mirrored in the site’s freedom of motion. Motions along the direction of positive CP are stiff and high frequency, while those along the direction of negative CP are much more fluid.

**Figure 9:**
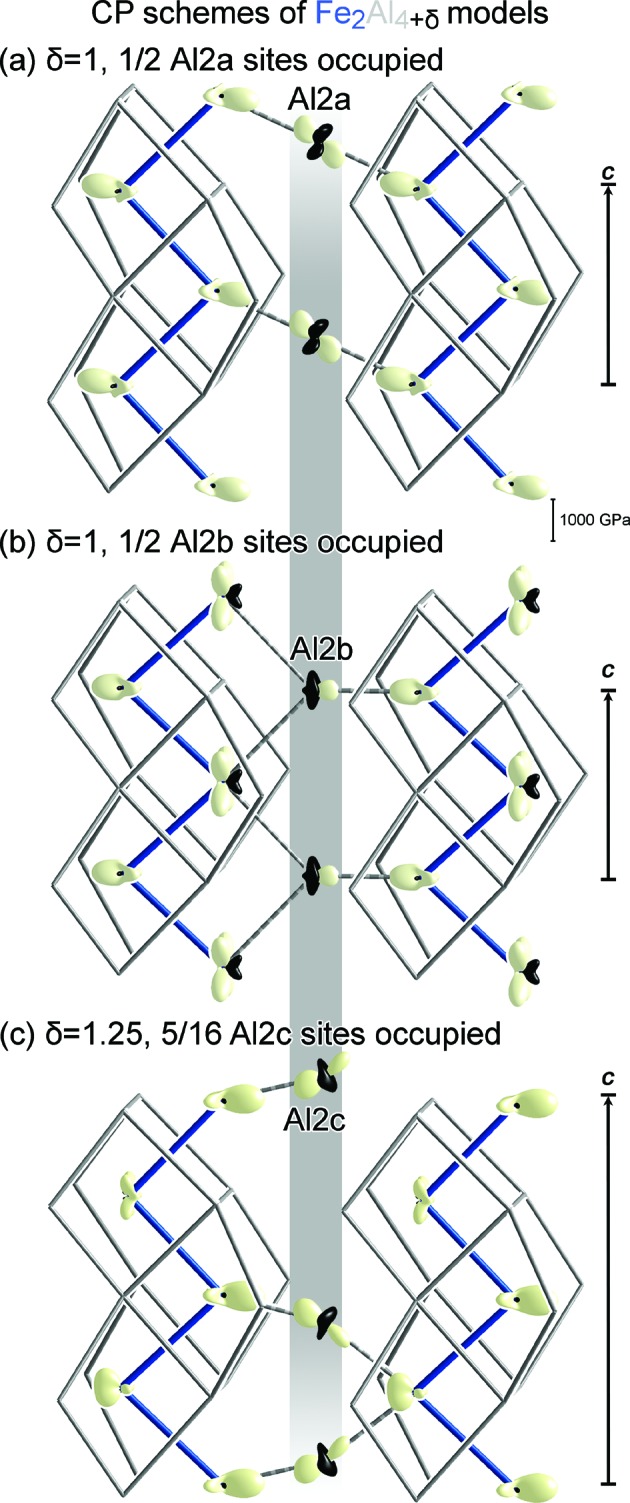
DFT-CPs underlying channel formation in Fe_2_Al_5_. The CPs experienced by the Fe and Al2 atom sites are shown in three ordered models: (*a*) Model 2, (*b*) Model 3 and (*c*) Model 1. A persistent feature is the presence of negative CP along the Al2 channel.

In Fig. 9 ▸, we shift our attention to the CP features for this region of the structure with other Al2 channel occupation patterns. In our next model [Fig. 9 ▸(*b*), Model 3], Al atoms are placed on every other Al2b site. One large positive CP is present on each of these atoms, oriented towards the nearest Fe neighbor. Although the Al2b site has shifted from the center of the channel (where the Al2a atoms are held) in response to this Fe atom, the growth of negative Al–Al CPs appears to prevent full relief of the Fe–Al positive CP. As in the previous model (Fig. 9 ▸ *a*), negative CPs are also directed perpendicular to the Fe–Al contacts, with a substantial component along the direction of diffuse electron density in the experimental structure.

The role of the Al2c site in the structural solution as a bridge between the Al2a and Al2b sites is reflected by its CP scheme in the more complicated superstructure of Model 1 (Fig. 9 ▸ *c*), where 5/16 of the Al2c sites are occupied. Yet again, strong positive CPs are directed towards the nearest Fe neighbors, while negative CPs are directed along the local path of the channel, and the distribution appears as an intermediate between the Al2a and Al2b CP schemes.

**Figure 10:**
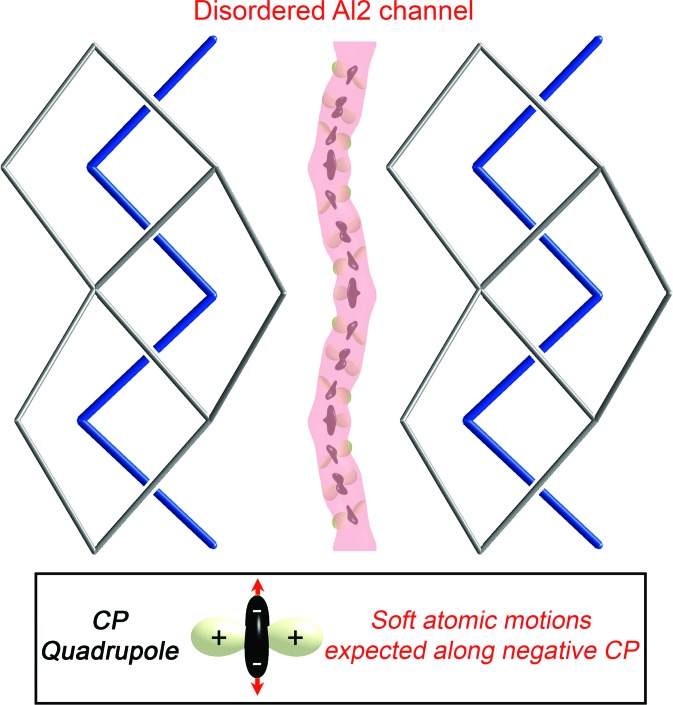
Placement of the Al2 CP distributions from the various ordered models within the experimental Fe_2_Al_5_ structure. A Fourier electron-density isosurface for the disordered region is shown for ρ = 5.0 e Å^−3^.

In all three models, then, the placement of perpendicular positive and negative CP lobes on the Al2 atoms produces a feature strongly correlated with soft phonon modes, a CP quadrupole. Intriguingly, these motions would tend to guide the Al2a site into the Al2b site (via the Al2c site) and vice versa. Such a path is emphasized in Fig. 10 ▸, where Al2 CPs from these three models are plotted within an isosurface of the Fourier electron density for this region from the experimental diffraction data. The negative pressure features of the CP distributions align closely with the continuous path of the electron density, hinting at easy diffusion of Al atoms along the channel between the Fe zigzag chains. This scheme also provides a rationale for the facile motions of Al atoms along these channels observed in earlier phonon calculations (Mihalkovič & Widom, 2012 ▸).

Overall, the CP distributions of these ordered models of Fe_2_Al_5_ reveal CP quadrupoles on the Al2 sites which describe an undulating path along *c.* When we recall that optimizing the bonding scheme of this compound requires nonstoichiometry on the Al sublattice, this region of the structure appears ideally suited to accommodate it. The lack of a clear preference for any particular positioning of the Al atoms within the channel, except to maximize Fe–Al distances, anticipates the weak positional order along it. The ease with which atoms move along the path also means that they would have little trouble shifting in response to the addition or removal of another Al atom in the channel, accounting for the observed compositional range of the phase.

## 4. Conclusions   

This work was inspired by the curious disordered channels of Al atoms that appear in the high-temperature structure of Fe_2_Al_5_ and contribute to its promise as a thermoelectric material. After confirming an experimental model in which the Al atoms are disordered in an undulating pattern throughout the compound, we applied a series of theoretical methods to elucidate the role of this channel in stabilizing the structure. Using the raMO analysis, we traced the nonstoichiometry of the Al content to the desire by the Fe bonding network to achieve 18-electron configurations, following the 18 − *n* rule. DFT-CP schemes then revealed how the channels in the structure accommodate the Al2 disorder: the placement of these sites between zigzag chains of Fe atoms leads to CP quadrupoles that allow for soft motions threading Al atoms along the path defined by the Fe.

The way that electron counts and chemical pressures conspire to produce the disordered Al2 columns suggests an approach by which structures with similar order/disorder dichotomies could be induced in a wide range of intermetallic phases. Compounds that follow the 18 − *n* bonding scheme while exhibiting clusters, columns, networks or sheets of CP quadrupoles on some of the main group sites would be expected to be responsive to elemental substitutions affecting the electron concentration. In such cases, motions within the soft sublattices defined by the CP quadrupoles would provide a path by which main group atoms could be inserted or removed to maintain the 18 − *n* electron counts (with their sizes also of potential importance). We are looking forward to exploring how this idea might apply to such structural series as the Nowotny Chimney Ladder phases (Nowotny, 1970 ▸), and to the discovery of new intermetallic compounds.

## 5. Related literature   

The following references are cited in the supporting information: Bruker (2016 ▸), Rigaku (2015 ▸), von Goldbeck (1982 ▸), Grin *et al.* (1994 ▸), Petříček *et al.* (2014 ▸), Sheldrick (1996 ▸, 2015*a* ▸,*b* ▸).

## Supplementary Material

Crystal structure: contains datablock(s) I, II, III, IV. DOI: 10.1107/S2053273318017461/gv5002sup1.cif

Structure factors: contains datablock(s) I. DOI: 10.1107/S2053273318017461/gv5002Isup2.hkl

Structure factors: contains datablock(s) II. DOI: 10.1107/S2053273318017461/gv5002IIsup3.hkl

Structure factors: contains datablock(s) III. DOI: 10.1107/S2053273318017461/gv5002IIIsup4.hkl

Structure factors: contains datablock(s) IV. DOI: 10.1107/S2053273318017461/gv5002IVsup5.hkl

Experimental and computational details. DOI: 10.1107/S2053273318017461/gv5002sup6.pdf

CCDC references: 1880447, 1880448, 1880449, 1880452
