# Supplementary material for: Principles of weakly ordered domains in intermetallics: the cooperative effects of atomic packing and electronics in Fe2Al5
Source: Acta Crystallogr A Found Adv. 2019 Feb 21;75(Pt 2):297–306. doi: 10.1107/S2053273318017461 (PMC6396395; doi:10.1107/S2053273318017461)
Supplement: Supplementary file 6 [file a-75-00297-sup6.pdf]

Acta Cryst A FOUNDATIONS ADVANCES

Volume 75 (2019)

Supporting information for article:

Principles of Weakly Ordered Domains in Intermetallics: The Cooperative Effects of Atomic Packing and Electronics in Fe2Al5

Anastasiya I. Vinokur, Katerina P. Hilleke and Daniel C. Fredrickson

S1. Synthetic Results

The synthesis of Fe_2Al_5 for our structural analysis required an iterative process. Initial attempts targeting the $Fe_2Al_{5.6}$ stoichiometry from the prior single crystal refinement (Burkhardt *et al.*, 1994) resulted in nearly phase pure samples of the neighboring Al-rich phase, Fe_4Al_{13} (Grin *et al.*, 1994). In our consecutive experiments, the Al content was lowered until a nearly phase pure sample of the target phase was observed for a loading ratio of 1 Fe:2.7 Al. The purity and the identity of the sample were confirmed by comparing the experimental powder X-ray diffraction with the calculated peak positions for the Fe_2Al_5 structure. All of the major peaks matched the previously reported structure, while some of the minor peaks were attributed to a small amount of an $FeAl_3$ impurity. The homogeneity of the sample was verified by Back Scattered Electron (BSE) images, which showed a single phase in the sample.

Since the reports of Al content have varied over the years, and the phase diagram shows a homogeneity range for the phase of about 70-73 at% Al concentration (Goldbeck, 1982), it was imperative to determine the Fe:Al ratio independently of the single crystal experiments. Energy Dispersive X-ray Spectroscopy (EDS) measurements confirmed that the synthesized sample was free from elemental impurities and the qualitative Fe:Al ratio of ~2:5. Wavelength Dispersive X-ray Spectroscopy (WDS) was used to more quantitatively determine the stoichiometry, as its use of standards helps eliminate environmental factors that could influence the amount of Al detected. From WDS, the phase was measured to have a composition of $Fe_{1.0(2)}Al_{2.62(11)}$.

S2. Single crystal X-ray diffraction experiments

Crystals were picked from the crushed samples and were first analyzed at room temperature with an Oxford Diffraction Xcalibur E diffractometer with a Mo Kα (λ=0.71073 Å) sealed-tube X-ray source. Additional single crystal datasets were collected with the Oxford Cryojet HT accessory at 105 K, 150 K, and 400 K to examine the possibility of superstructure formation resulting from the changes in the disordered Al positions. For the room temperature data set, the run list consisted of ω scans chosen to cover a full sphere of reciprocal space out to a resolution of 0.8 Å. The scans were taken with a 0.6° step width and a 120 second exposure time at a crystal-to-detector distance of 70 mm. The run list for the full experiment at 105 K was also based on ω scans, this time with the same step width but an 80 second exposure time and detector distance of 50mm, chosen to cover a full sphere out to 0.8 Å resolution. A step width of 0.5° and exposure time of 80 seconds were used for additional data collections at 150 K and 400 K. The creation of the run lists and the processing of the frame data were performed with the CrysalisPro ver. 171 software (*CrysAlisPro Software System, Version 171.37.35*, 2015).

For the final variable temperature datasets, the data were collected with a Bruker Quazar APEX2 diffractometer with a Mo Kα IμS microfocus X-ray source (λ=0.71073 Å). The datasets were collected at 100 K, 150 K, 300 K, and 400 K. The run list consisted of ω and ϕ scans with 0.7º step size and exposure time of 40 seconds, designed to cover a full sphere to a resolution of 0.7 Å. The Apex III software package (Bruker, 2016) was used for data collection and integration. For absorption correction and data reduction SADABS (Sheldrick, 1996) and XPREP were used, respectively.

Analysis of the room temperature dataset revealed an orthorhombic unit cell with unit cell parameters of a=7.5660(3) Å, b=6.4117(2) Å, and c=4.22350(18) Å. The observed systematic absences in the reciprocal space reconstructions were consistent with space group *Cmcm* and the subsequent structure solution and refinement confirmed the correct assignment of the space group symmetry.

An intrinsic phasing algorithm as implemented in ShelXT (Sheldrick, 2015b) was used to solve the single crystal structure, yielding 2 symmetry-distinct atomic positions. The three positions to model the disordered Al site were obtained from the Fourier difference map. The occupancies of the three disordered Al sites were refined freely, while their thermal displacement parameters were constrained to be the same. The resulting combined occupancy was less than 100%, indicating that the electron density column is only partially occupied. The structure was refined on F^2 using ShelXL (Sheldrick, 2015a). Atoms Fe1 and Al1 were refined anisotropically, while Al2a, Al2b, and Al2c were refined isotropically. The final refinement converged to R(I>3σ)=1.32 for the 300 K dataset. The largest peaks in the Fourier difference map corresponded to maximum and minimum densities of 0.36 electrons/Å^3 and -0.27 electrons/Å^3, respectively. The formula refined to $Fe_2Al_{5.5}$. Maps of the Fourier electron density were constructed with the Jana2006 program (Petříček *et al.*, 2014).

The data collected at 100 K, 150 K and 400 K could also be indexed with an orthorhombic unit cell with similar parameters to the room temperature data (Figure S1), with the trends being interpretable in terms of thermal expansion. The systematic absences in the three datasets were consistent with *Cmcm* space group. The solutions and refinements yielded structures similar to the room temperature results. The same model was applied to the solution and refinement of the 100 K, 150 K, and 400 K data sets.

The crystal structure refinement details for the four datasets are given in Table S1. The refined atomic coordinates, atomic displacement parameters, and selected interatomic distances are given in Tables S2-S13.

Table S1 Crystallographic data for Fe_2Al_5

Chemical formula	$Fe_2Al_{5.5}$
WDS composition	$Fe_{2.0(4)}Al_{5.2(2)}$

Crystal dim. (mm^3)	0.106× 0.093 × 0.033			
Crystal color	Metallic gray			
Radiation source, λ (Å)	Mo Kα, (0.71073 Å)			
Absorption correction	Multi-scan			
Data collection temp.	100K	150K	300K	400K
Pearson Symbol		*o*C15		
Space group		*Cmcm* (No. 63)		
a (Å)	7.638(2)	7.642(2)	7.651(2)	7.660(2)
b (Å)	6.3949(18)	6.3978(18)	6.4087(18)	6.4183(18)
c (Å)	4.2098(14)	4.2111(14)	4.2173(14)	4.2224(14)
Cell volume ($Å^3$)	205.64(11)	205.88(11)	206.78(11)	207.59(11)
Refined Composition	$Fe_2Al_{5.508(5)}$	$Fe_2Al_{5.506(5)}$	$Fe_2Al_{5.504(6)}$	$Fe_2Al_{5.503(6)}$
Calc. density (g/cm^3)	4.205	4.1980	4.179	4.163
Absorption coef. (mm^{-1})	8.016	8.005	7.970	7.939
θ_{min}, θ_{max}	4.16, 30.62	4.15, 30.62	4.15, 30.43	4.14, 30.53
Number of reflections	1862	1872	1864	1860
R_{int} (I> 3σ)	1.91	2.12	1.95	1.90
Unique refl. (I > 3σ)	188	189	187	189
Number of parameters	18	18	18	18
R (I > 3σ), R_w (I > 3σ)	1.18, 3.17	1.32, 3.44	1.32, 3.47	1.33, 3.43
R(all), R_w(all)	1.20, 3.17	1.33, 3.44	1.33, 3.47	1.36, 3.44
S (I >3σ)	1.156	1.101	1.183	1.158
$\Delta\rho_{max}$, $\Delta\rho_{min}$ ($e^-/Å^3$)	0.35, -0.30	0.43, -0.31	0.36, -0.27	0.26, -0.27

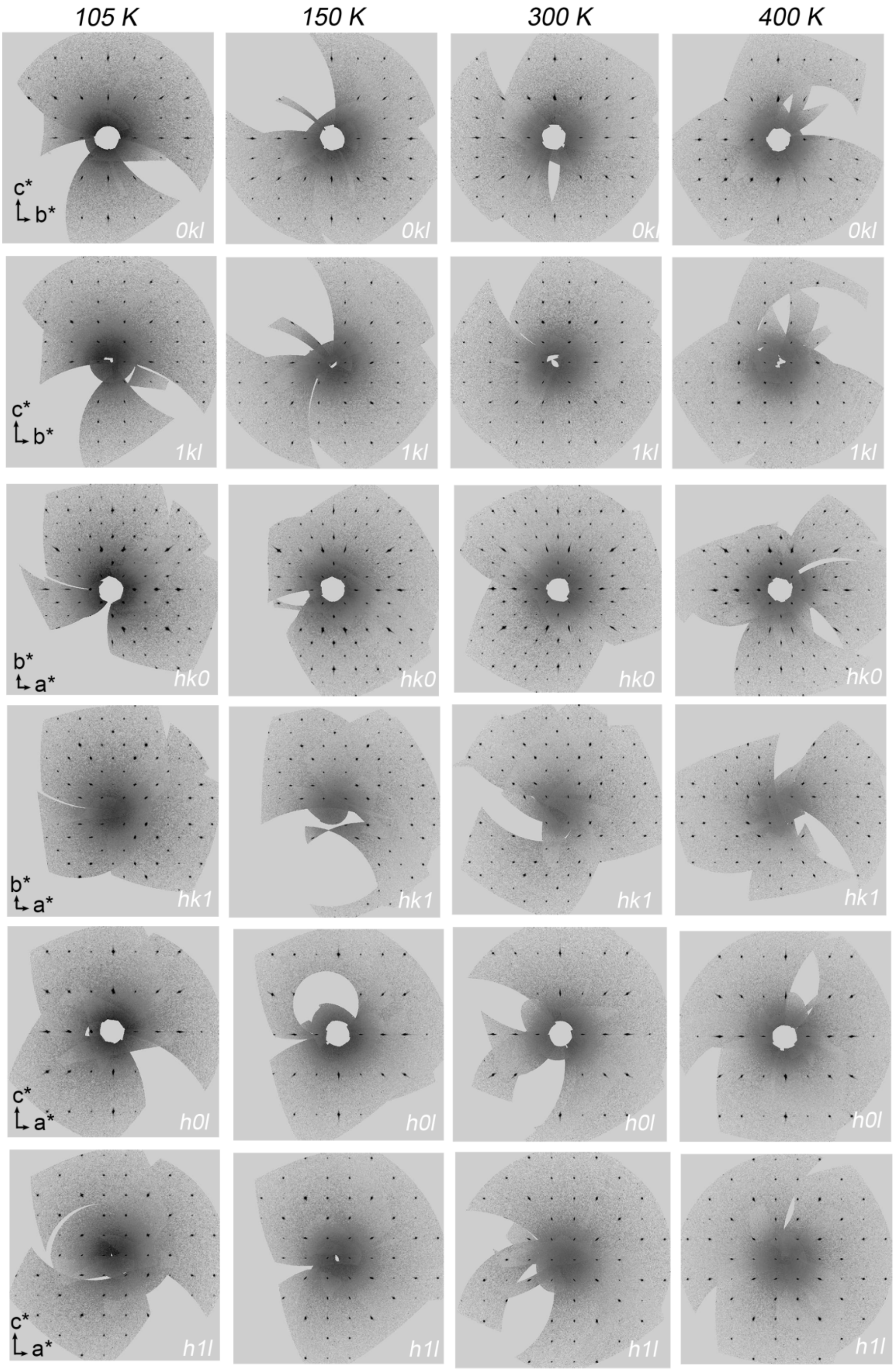

Figure S1 Reciprocal space reconstructions of 0*kl*, 1*kl*, *hk*1, *h*0*l*, and *h*1*l* layers.

Table S2 Atomic coordinates and displacement parameters for $Fe_2Al_{5.5}$ at 100K

Site	Wyckoff Position	*x*	*y*	*z*	U_{equiv}/U_{iso}	Occupancy
Fe1	4c	0.5000	0.82803(4)	0.2500	0.00633(11)	1.0
Al1	8g	0.31298(7)	0.14790(8)	0.2500	0.01036(13)	1.0
Al2a	4a	0.5000	0.5000	0.5000	0.0064(5)	0.251(4)
Al2b	4c	0.5000	0.4617(6)	0.2500	0.0064(5)	0.184(4)
Al2c	8f	0.5000	0.5230(5)	0.6214(18)	0.0064(5)	0.1594(15)

Table S3 Anisotropic atomic displacement parameters for $Fe_2Al_{5.5}$ at 100K

Site	U_{11}	U_{22}	U_{33}	U_{12}	U_{13}	U_{23}
Fe1	0.00414(17)	0.00602(17)	0.00883(17)	0.00000	0.00000	0.00000
Al1	0.0083(2)	0.0165(3)	0.0063(2)	0.00000	0.00000	0.00726(18)

Table S4 Selected interatomic distances for $Fe_2Al_{5.5}$ at 100K

Site	Neighbor	Distance (Å)
Fe1	Al1(×3)	2.5485(7)
	Al1(×2)	2.4950(7)
	Al2a(×2)	2.3469(6)
	Al2b	2.343(4)
	Al2c(×2)	2.309(3)
	Al2c(×2)	2.500(5)
Al1	Al1(×2)	2.6573(8)
	Al2b	2.463(3)
	Al2b	2.6709(19)
	Al2c(×2)	2.601(3)
	Al2c(×2)	2.6837(19)
Al2a	Al2a(×2)	2.1049(7)
	Al2c(×2)	1.0806(10)
Al2b	Al2b(×2)	2.1613(19)
	Al2c(×2)	1.612(8)
	Al2c(×2)	0.550(8)
	Al2c	2.675(7)
Al2c	Al2c(×2)	2.1253(11)
	Al2c(×2)	1.064(15)

Table S5 Atomic coordinates and displacement parameters for $Fe_2Al_{5.5}$ at 150K

Site	Wyckoff Position	*X*	*y*	*z*	U_{equiv}/U_{iso}	Occupancy
Fe1	4c	0.5000	0.82803(5)	0.2500	0.00708(12)	1.0
Al1	8g	0.31293(7)	0.14788(8)	0.2500	0.01129(15)	1.0
Al2a	4a	0.5000	0.5000	0.5000	0.0073(5)	0.254(5)
Al2b	4c	0.5000	0.4613(7)	0.2500	0.0073(5)	0.179(4)
Al2c	8f	0.5000	0.5232(5)	0.623(2)	0.0073(5)	0.1599(16)

Table S6 Anisotropic atomic displacement parameters for $Fe_2Al_{5.5}$ at 150K

Site	U_{11}	U_{22}	U_{33}	U_{12}	U_{13}	U_{23}
Fe1	0.00473(18)	0.00702(18)	0.00949(18)	0.00000	0.00000	0.00000
Al1	0.0092(3)	0.0178(3)	0.0069(2)	0.00000	0.00000	0.00718(19)

Table S7 Selected interatomic distances for $Fe_2Al_{5.5}$ at 150K

Site	Neighbor	Distance (Å)
Fe1	Al1(×3)	2.5496(7)
	Al1(×2)	2.4962(8)
	Al2a(×2)	2.3479(6)
	Al2b	2.346(4)
	Al2c(×2)	2.310(3)
	Al2c(×2)	2.505(5)
Al1	Al1(×2)	2.6582(9)
	Al2b	2.463(4)
	Al2b	2.673(2)
	Al2c(×2)	2.599(4)
	Al2c(×2)	2.683(2)
Al2a	Al2a(×2)	2.1056(7)
	Al2b(×2)	1.0815(11)
Al2b	Al2b(×2)	2.163(2)
	Al2c(×2)	1.621(9)
	Al2c(×2)	0.542(8)

	Al4	2.668(8)

Table S8 Atomic coordinates and displacement parameters for $Fe_2Al_{5.5}$ at 300K

Site	Wyckoff Position	*X*	*y*	*z*	U_{equiv}/U_{iso}	Occupancy
Fe1	4c	0.5000	0.82792(5)	0.2500	0.00860(12)	1.0
Al1	8g	0.31295(8)	0.14786(8)	0.2500	0.01332(15)	1.0
Al2a	4a	0.5000	0.5000	0.5000	0.0093(5)	0.252(5)
Al2b	4c	0.5000	0.4614(7)	0.2500	0.0093(5)	0.176(5)
Al2c	8f	0.5000	0.5232(5)	0.623(2)	0.0093(5)	0.1621(17)

Table S9 Anisotropic atomic displacement parameters for $Fe_2Al_{5.5}$ at 300K

Site	U_{11}	U_{22}	U_{33}	U_{12}	U_{13}	U_{23}
Fe1	0.00634(18)	0.00811(18)	0.01135(19)	0.00000	0.00000	0.00000
Al1	0.0112(3)	0.0197(3)	0.0090(3)	0.00000	0.00000	0.0076(2)

Table S10 Selected interatomic distances for $Fe_2Al_{5.5}$ at 300K

Site	Neighbor	Distance (Å)
Fe1	Al1(×3)	2.5531(7)
	Al1(×2)	2.5004(8)
	Al2a(×2)	2.3512(6)
	Al2b	2.349(5)
	Al2c(×2)	2.313(3)
	Al2c(×2)	2.508(5)
Al1	Al1(×2)	2.6624(9)
	Al2b	2.467(4)
	Al2b	2.676(2)
	Al2c(×2)	2.603(4)
	Al2c(×2)	2.687(2)
Al2a	Al2a(×2)	2.1087(7)
	Al2b(×2)	1.0830(11)
Al2b	Al2b(×2)	2.166(2)
	Al2c(×2)	1.623(9)
	Al2c(×2)	0.544(8)

	Al2c	2.673(8)
Al2c	Al2c(×2)	2.1295(12)
	Al2c(×2)	1.081(17)

Table S11 Atomic coordinates and displacement parameters for $Fe_2Al_{5.5}$ at 400K

Site	Wyckoff Position	*X*	*y*	*z*	U_{equiv}/U_{iso}	Occupancy
Fe1	4c	0.5000	0.82782(5)	0.2500	0.00996(12)	1.0
Al1	8g	0.31294(8)	0.14776(9)	0.2500	0.01514(15)	1.0
Al2a	4a	0.5000	0.5000	0.5000	0.0111(5)	0.249(5)
Al2b	4c	0.5000	0.4609(8)	0.2500	0.0111(5)	0.175(5)
Al2c	8f	0.5000	0.5225(6)	0.622(2)	0.0111(5)	0.1637(19)

Table S12 Anisotropic atomic displacement parameters for $Fe_2Al_{5.5}$ at 400K

Site	U_{11}	U_{22}	U_{33}	U_{12}	U_{13}	U_{23}
Fe1	0.00781(18)	0.00916(18)	0.01290(19)	0.00000	0.00000	0.00000
Al1	0.0130(3)	0.0218(3)	0.0106(3)	0.00000	0.00000	0.0079(2)

Table S13 Selected interatomic distances for $Fe_2Al_{5.5}$ at 400K

Site	Neighbor	Distance (Å)
Fe1	Al1(×3)	2.5563(7)
	Al1(×2)	2.5039(8)
	Al2a(×2)	2.3540(6)
	Al2b	2.355(5)
	Al2c(×2)	2.312(4)
	Al2c(×2)	2.512(6)
Al1	Al1(×2)	2.6663(9)
	Al2b	2.468(4)
	Al2b	2.680(2)
	Al2c(×2)	2.612(4)
	Al2c(×2)	2.689(2)
Al2a	Al2a(×2)	2.1112(7)
	Al2b(×2)	1.0850(12)
Al2b	Al2b(×2)	2.170(2)

	Al2c(×2)	1.623(9)
	Al2c(×2)	0.549(9)
	Al2c	2.679(9)
Al2c	Al2c(×2)	2.1309(12)
	Al2c(×2)	1.073(19)

S3. Powder X-ray Diffraction

The phase analysis with powder X-ray diffraction was performed on the manually ground powder of the crushed sample that was then mounted onto a glass fiber with vacuum grease. A Rigaku Rapid II diffractometer with Mo Kα radiation (λ=0.71073 Å) equipped with a curved image plate detector was used for all powder X-ray diffraction data sets. The averaging over the frames to create I vs. 2θ profiles was performed with the 2DP Pattern Integration software for the 2θ range 2° to 45° with a step size 0.02°. All major peaks agreed well with calculated pattern for the previously reported Fe_2Al_5 structure, as is shown in Figure S2.

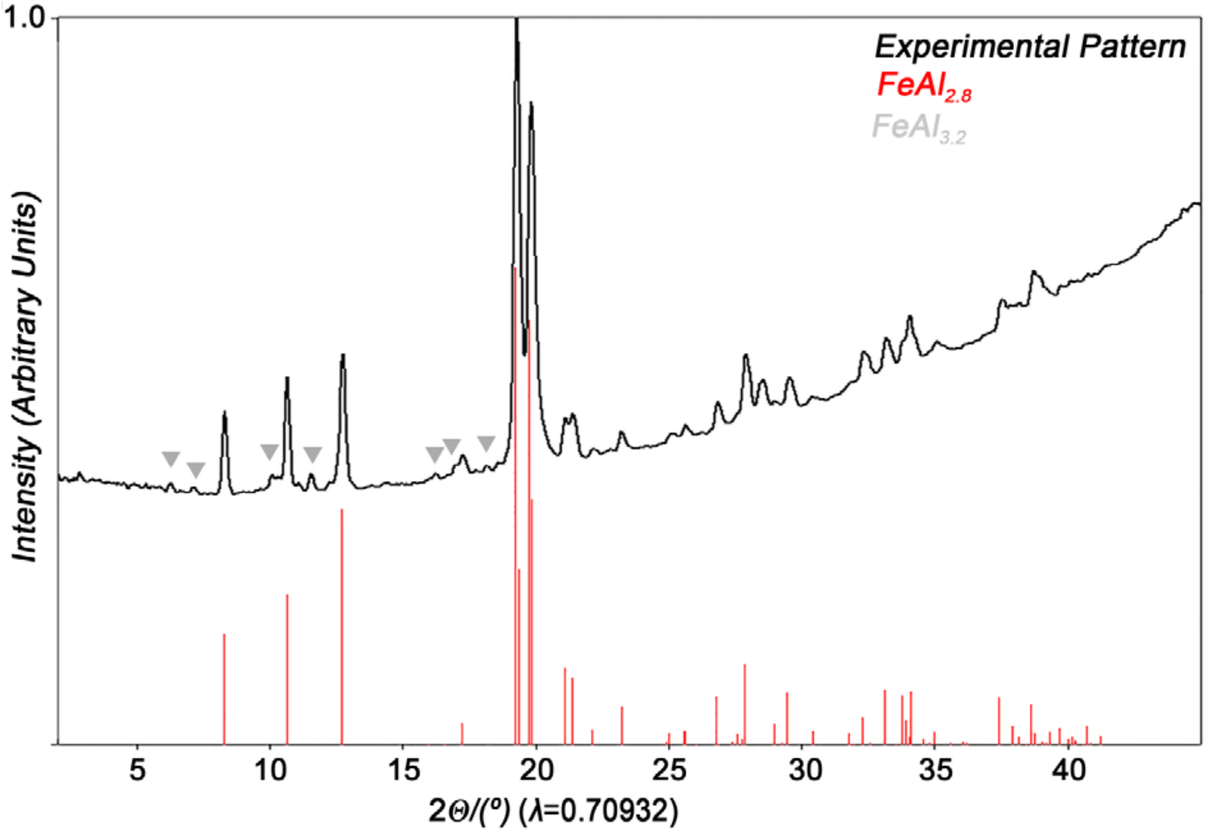

Figure S2 Experimental powder X-ray diffraction pattern. The label "$FeAl_{2.8}$" refers to the Fe_2Al_5 phase.

S4. Elemental Analysis with Energy Dispersive X-ray Spectroscopy (EDS)

The energy dispersive X-ray spectroscopy (EDS) measurements were performed on samples prepared by suspending crushed pieces of the reaction product in epoxy. The sample was hand-polished with a diamond lapping film to create a flat surface and was then carbon coated. The elemental analysis was carried out with a Hitachi S-3100N Scanning Electron Microscope equipped with an EDS probe (Voltage=15 keV). The sample appeared homogeneous with the single phase (Figure S3) whose composition was measured to be $Fe_{2.069(19)}Al_{4.930(18)}$ (average of 10 points). No elemental impurities other than O and C (from the coating) were observed.

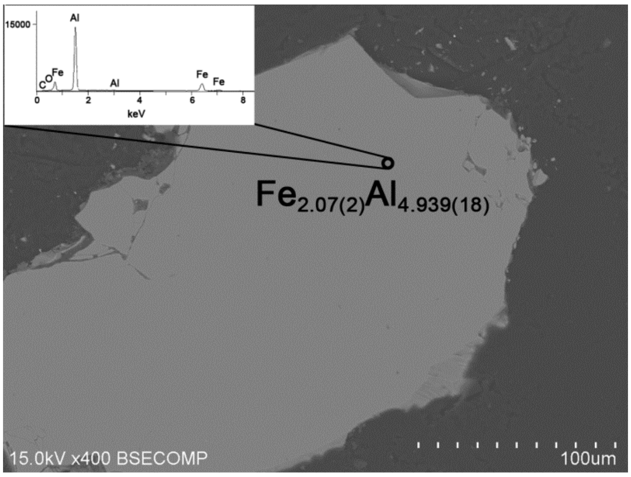

Figure S3 Back-scattered electron image of the sample shows only one phase, as evidenced by the observed single shade of gray in the image.

S5. Elemental Analysis with Wavelength Dispersive X-ray Spectroscopy (WDS)

Due to the key role that the Al content plays in our electronic structure discussion, the elemental composition was further investigated using Wavelength Dispersive X-ray Spectroscopy (WDS). To prepare the sample, powder of the reaction product was suspended in conducting epoxy. The surface was polished using polycrystalline diamond suspension spread over a polishing wheel and carbon coated. CAMECA SX51 Electron Probe Microanalyzer (Voltage=15 eV, Current=20 nA) was used for the elemental analysis. An $FeAl_{3.10}$ standard was used. One phase was observed in the sample with a composition of $Fe_{2.0(4)}Al_{5.2(2)}$ (average of 10 measurement points).

S6. Computational Details and Optimized Geometries for the raMO analysis

Table S14 Al channel occupation patterns for theoretical structure models.[a]

Model	2a	2c	2b	2c	2a	2c	2b	2c	2a	2c	2b	2c	2a	2c	2b	2c
Model 1																
column 1		●				●						●				
column 2		●										●				
Model 2																
column 1					●								●			
column 2					●								●			
Model 3																
column 1			●								●					
column 2			●								●					
Model 4[b]																
column 1		●				●						●				
column 2				●						●						
Model 5																
column 1	●								●							
column 2					●								●			

[a] Al2a refers to sites with z= 0, 0.50, 1.00, and 1.50 reading from left to right across the table, with reference to the average cell of Fe_2Al_5. Al2b refers to sites with z= 0.25, 0.75, 1.25, and 1.75 reading from left to right. Al2c sites correspond to atoms that lie between these special points, though not necessarily at the exact points given in the experimental refined structure.

[b] Upon geometrical optimization in VASP, this model converged on a geometry essentially identical to Model 1. It is then not considered further.

Table S15 DFT-optimized (VASP, GGA) geometry for Model 1 ($Fe_2Al_{5.25}$).

Cell Vectors	*x* (Å)	*y* (Å)	*z* (Å)

**a** (Å)	7.50730887168113457	0.0	0.0
**b** (Å)	0.0	6.4022835217282674	-0.0020595018067675
**c** (Å)	0.0	-0.0027044037439530	8.4055356542294550
	x	*y*	*z*
Fe	0.5000000000000000	0.8177268479162928	0.1299730207628850
Fe	0.5000000000000000	0.8453147342087978	0.6278795982030294
Fe	0.5000000000000000	0.1659512708310177	0.3759622929357095
Fe	0.5000000000000000	0.1648509315349287	0.8830646198195269
Fe	0.0000000000000000	0.3215668421017429	0.1288041209064729
Fe	0.0000000000000000	0.3303958197854167	0.6297541770565580
Fe	0.0000000000000000	0.6720704840689798	0.3882795908173120
Fe	0.0000000000000000	0.6723645077767849	0.8686851444096846
Al	0.3217721784985529	0.1549254310623098	0.1291250041066531
Al	0.3257762495050863	0.1720022405122511	0.6296520792025114
Al	0.6866739814137159	0.8569290853681012	0.3882740189329492
Al	0.6848483101742543	0.8528706627294723	0.8695753071989597
Al	0.6782278215014473	0.1549254310623098	0.1291250041066531
Al	0.6742237504949137	0.1720022405122511	0.6296520792025114
Al	0.3133260185862840	0.8569290853681012	0.3882740189329492
Al	0.3151516898257457	0.8528706627294723	0.8695753071989597
Al	0.8161781699979922	0.6544147432047405	0.1292693027360805
Al	0.7802090109429147	0.6201224307717133	0.6282850791019219
Al	0.1827222370771223	0.3462480239432285	0.3810522830798195
Al	0.1839589651213630	0.3493240164236130	0.8772991123036962
Al	0.1838218300020078	0.6544147432047405	0.1292693027360805
Al	0.2197909890570855	0.6201224307717133	0.6282850791019219
Al	0.8172777629228779	0.3462480239432285	0.3810522830798195
Al	0.8160410348786371	0.3493240164236130	0.8772991123036962
Al	0.5000000000000000	0.5141750561316951	0.9569155720736267

Al	0.0000000000000000	0.0113152210115852	0.9646825271376616
Al	0.0000000000000000	0.9508699964234433	0.6266370507552911
Al	0.5000000000000000	0.5190741652433334	0.3144181277788695
Al	0.0000000000000000	0.0083508969351109	0.2862791360181945
Total Energy: -5.277 eV/atom			

Table S16 DFT-optimized (VASP, GGA) geometry for Model 2 (Fe_2Al_5).

Cell Vectors	*x* (Å)	*y* (Å)	*z* (Å)
**a** (Å)	7.3881076145399893	0.0	0.0
**b** (Å)	0.0	6.4853063865299507	-0.1682883068546966
**c** (Å)	0.0	-0.2196602326501776	8.4220588198942217
	x	*y*	*z*
Fe	0.4999999990000035	0.8372788234583258	0.1204219977872812
Fe	0.4999999990000035	0.8372788234583258	0.6204220257872837
Fe	0.4999999990000035	0.1627212015416798	0.3795780302127140
Fe	0.4999999990000035	0.1627212015416798	0.8795779392127170
Fe	0.0000000000000000	0.3372788114583247	0.1204219977872812
Fe	0.0000000000000000	0.3372788114583247	0.6204220257872837
Fe	0.0000000000000000	0.6627212135416737	0.3795780302127140
Fe	0.0000000000000000	0.6627212135416737	0.8795779392127170
Al	0.3173457944911697	0.1565647715592769	0.1276052600207477
Al	0.3173457944911697	0.1565647715592769	0.6276052880207499
Al	0.6826542035088301	0.8434352524407250	0.3723947679792476
Al	0.6826542035088301	0.8434352524407250	0.8723946769792508
Al	0.6826542035088301	0.1565647715592769	0.1276052600207477
Al	0.6826542035088301	0.1565647715592769	0.6276052880207499
Al	0.3173457944911697	0.8434352524407250	0.3723947679792476
Al	0.3173457944911697	0.8434352524407250	0.8723946769792508

Al	0.8173457934911661	0.6565647835592779	0.1276052600207477
Al	0.8173457934911661	0.6565647835592779	0.6276052880207499
Al	0.1826542045088337	0.3434352404407240	0.3723947679792476
Al	0.1826542045088337	0.3434352404407240	0.8723946769792508
Al	0.1826542045088337	0.6565647835592779	0.1276052600207477
Al	0.1826542045088337	0.6565647835592779	0.6276052880207499
Al	0.8173457934911661	0.3434352404407240	0.3723947679792476
Al	0.8173457934911661	0.3434352404407240	0.8723946769792508
Al	0.4999999990000035	0.5000000120000010	0.0000000000000000
Al	0.4999999990000035	0.5000000120000010	0.5000000280000023
Al	0.0000000000000000	0.0000000000000000	0.0000000000000000
Al	0.0000000000000000	0.0000000000000000	0.5000000280000023
Total Energy: -5.307 eV/atom			

Table S17 DFT-optimized (VASP, GGA) geometry for Model 3 (Fe_2Al_5).

Cell Vectors	*x* (Å)	*y* (Å)	*z* (Å)
**a** (Å)	7.5268888474	0.0	0.0
**b** (Å)	0.0	6.5470576286	0.0
**c** (Å)	0.0	0.0	4.0576219559
	x	*y*	*z*
Fe	0.0	0.334362	0.75
Fe	0.5	0.834362	0.75
Fe	0.0	0.674478	0.25
Fe	0.5	0.174478	0.25
Al	0.185836	0.348052	0.25
Al	0.685836	0.848052	0.25
Al	0.814047	0.642391	0.75
Al	0.314047	0.142391	0.75

Al	0.814164	0.348052	0.25
Al	0.314164	0.848052	0.25
Al	0.185953	0.642391	0.75
Al	0.685953	0.142391	0.75
Al	0.0	0.971973	0.75
Al	0.5	0.471973	0.75
Total Energy: -5.256 eV/atom			

Table S18 DFT-optimized (VASP, GGA) geometry for Model 5 (Fe_2Al_5).

Cell Vectors	*x* (Å)	*y* (Å)	*z* (Å)
**a** (Å)	7.4195734384313718	0.0	0.0
**b** (Å)	0.0	6. 4728088642862360	-0.0001404894428292
**c** (Å)	0.0	-0.0001790488355834	8. 3613263111077227
	x	*y*	*z*
Fe	0.0	0.334195	0.119487
Fe	0.5	0.834267	0.130535
Fe	0.0	0.665805	0.380513
Fe	0.5	0.165733	0.369465
Fe	0.0	0.334195	0.619487
Fe	0.5	0.834267	0.630535
Fe	0.0	0.665805	0.880513
Fe	0.5	0.165733	0.869465
Al	0.0	0.0	0.0
Al	0.0	0.0	0.5
Al	0.5	0.5	0.25
Al	0.5	0.5	0.75
Al	0.684277	0.153569	0.623858
Al	0.184328	0.653488	0.626051
Al	0.684277	0.846431	0.876142

Al	0.184328	0.346512	0.873949
Al	0.684277	0.153569	0.123858
Al	0.184328	0.653488	0.126051
Al	0.684277	0.846431	0.376142
Al	0.184328	0.346512	0.373949
Al	0.315724	0.846431	0.876142
Al	0.815672	0.346512	0.873949
Al	0.315724	0.153569	0.623858
Al	0.815672	0.653488	0.626051
Al	0.315724	0.846431	0.376142
Al	0.815672	0.346512	0.373949
Al	0.315724	0.153569	0.123858
Al	0.815672	0.653488	0.126051
Total Energy: -5.292 eV/atom			

Table S19 DFT-calibrated Hückel Parameters

Compound, RMS deviation[a]	Orbital	H_{ii}(eV)	c_1[b]	$\zeta_1(a_o^{-1})$	c_2[b]	$\zeta_2(a_o^{-1})$
Model 1 ($Fe_2Al_{5.25}$)	Fe 4s	-4.446	1.0000	2.3371	0.0000	0.0000
0.107 eV	Fe 4p	-3.598	1.0000	2.4117	0.0000	0.0000
	Fe 3d	-8.804	0.5680	5.9016	0.9633	2.2427
	Al 3s	-10.496	1.0000	2.1896	0.0000	0.0000
	Al 3p	-5.747	1.0000	1.9484	0.0000	0.0000
Model 2 (Fe_2Al_5)	Fe 4s	-5.608	1.0000	2.7893	0.0000	0.0000
0.119 eV	Fe 4p	-3.110	1.0000	2.5862	0.0000	0.0000
	Fe 3d	-8.701	0.5680	5.9016	0.9633	2.2805
	Al 3s	-10.292	1.0000	2.0994	0.0000	0.0000

Al 3p	-5.873	1.0000	1.9322	0.0000	0.0000

[a] Root-mean-squared deviation between the DFT and Hückel band energies up to ca. 1 eV above the E_F

[b] For the double-ζ d orbitals, the c_1 and c_2 coefficients are scaled for normalization inside the YAeHMOP program.

S7. Comparison of GGA-DFT and DFT-calibrated Hückel DOS Distributions, and additional DOS results

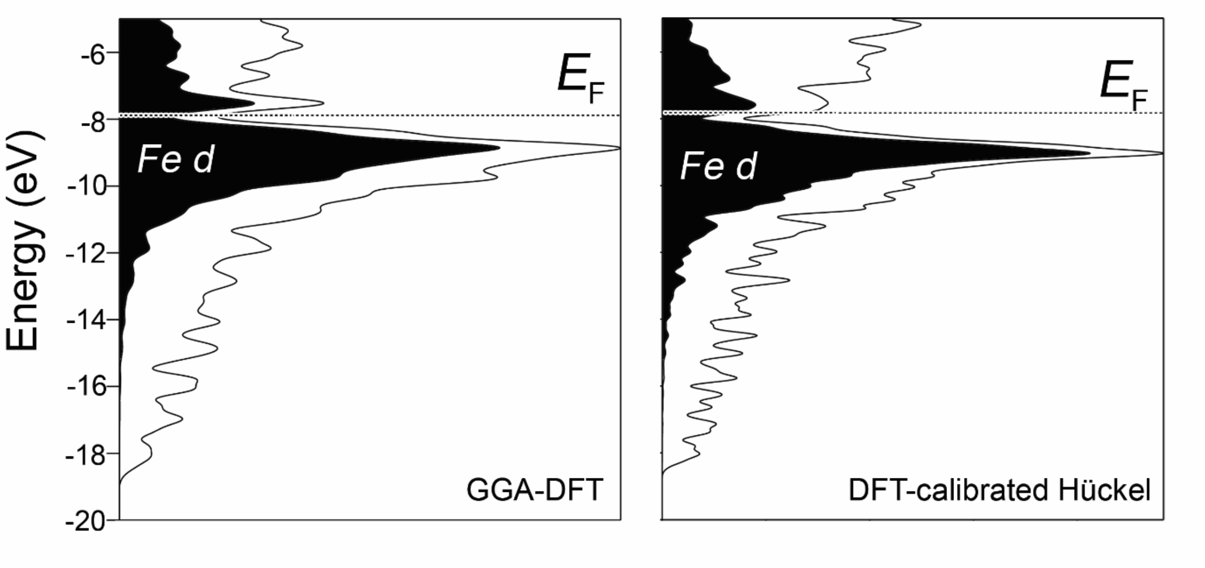

Figure S4 Electronic DOS distributions calculated for Model 1 ($Fe_2Al_{5.25}$) with GGA-DFT (left) and the best-fit Hückel model (right).

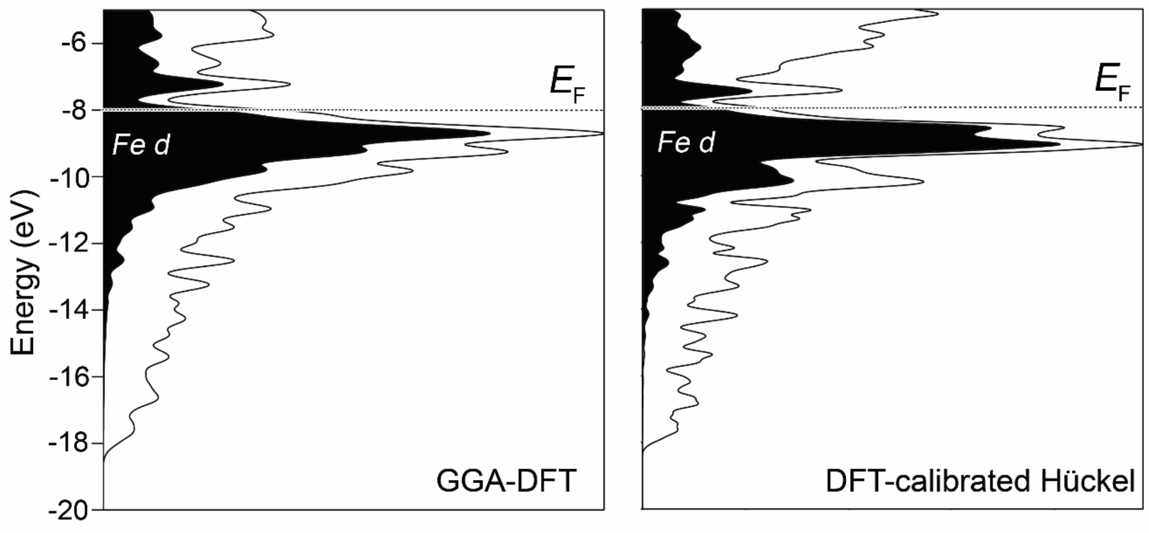

Figure S5 Electronic DOS distributions calculated for Model 2 (Fe_2Al_5) with GGA-DFT (left) and the best-fit Hückel model (right).

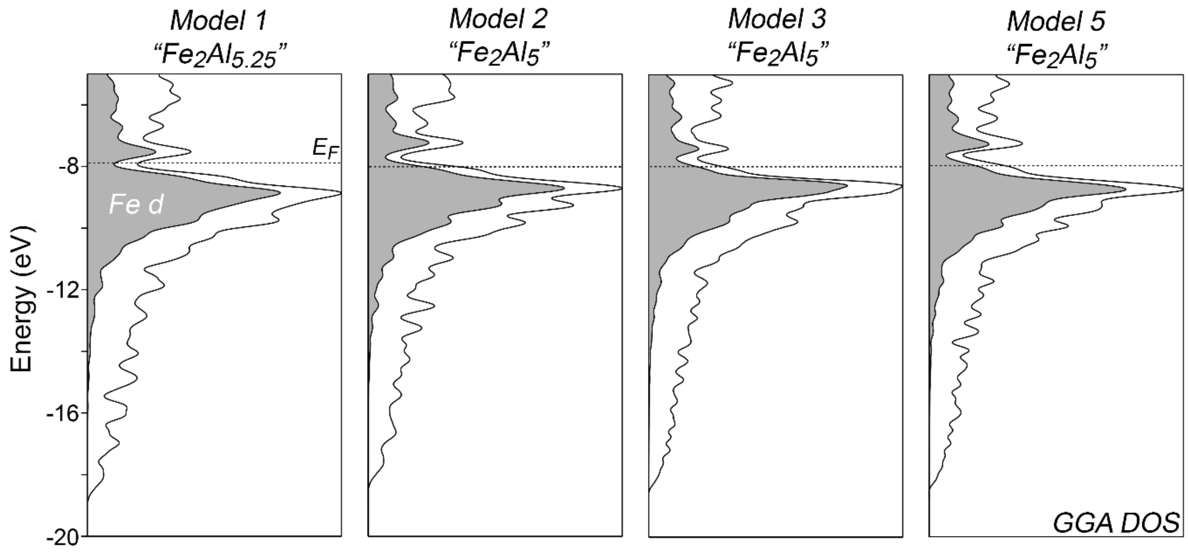

Figure S6 Comparison of electron DOS distributions for Models 1-3 and 5.

S8. raMO Results for Fe Atoms in "$Fe_2Al_{5.25}$" (Model 1)

raMOs and LC-raMOs for Fe4 are shown in Figures 5 and 6 of the main text.

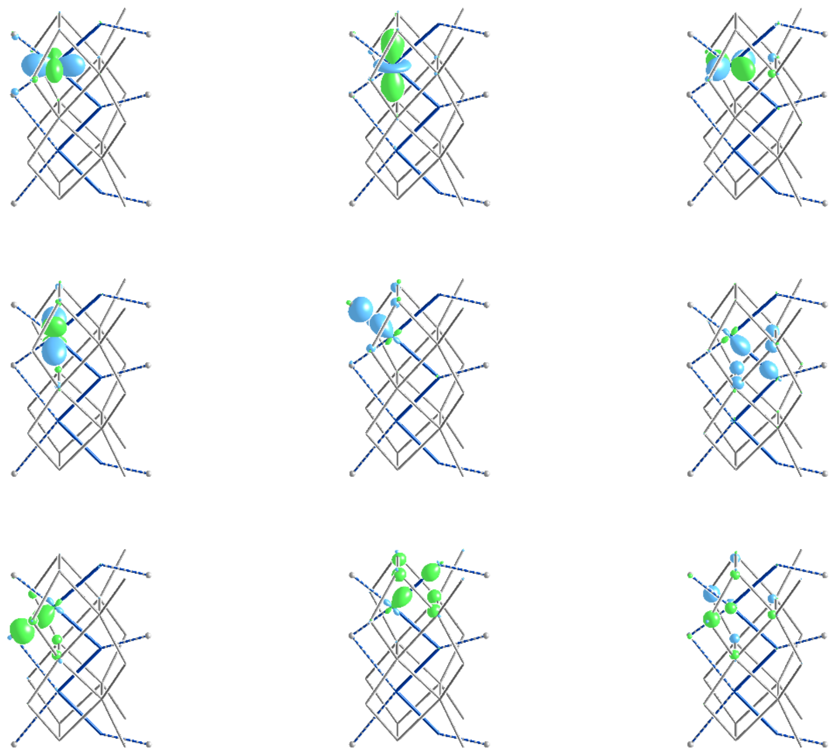

Figure S7 LC-raMO results for Fe1.

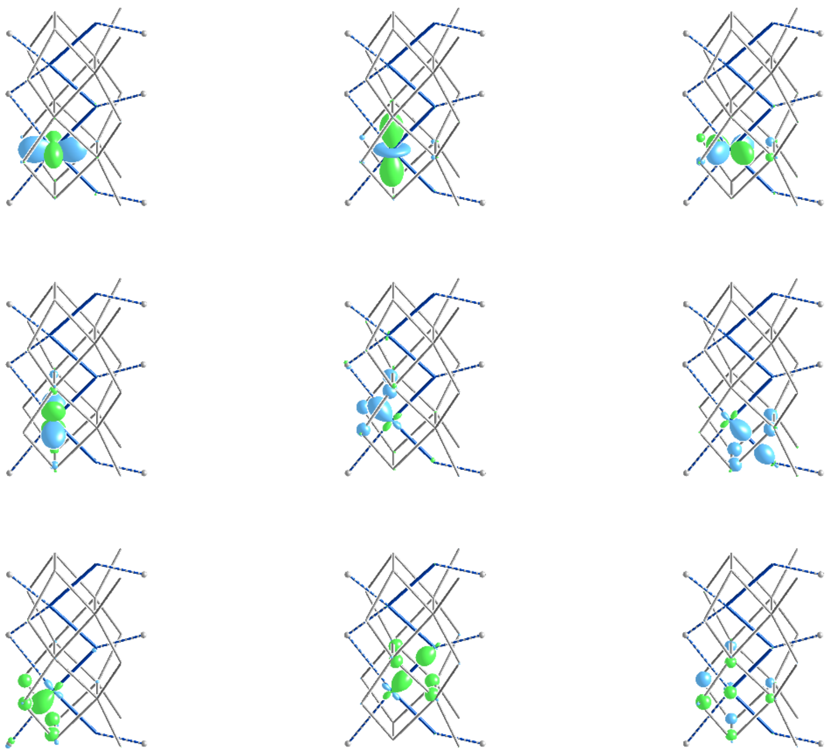

Figure S8 LC-raMO results for Fe2.

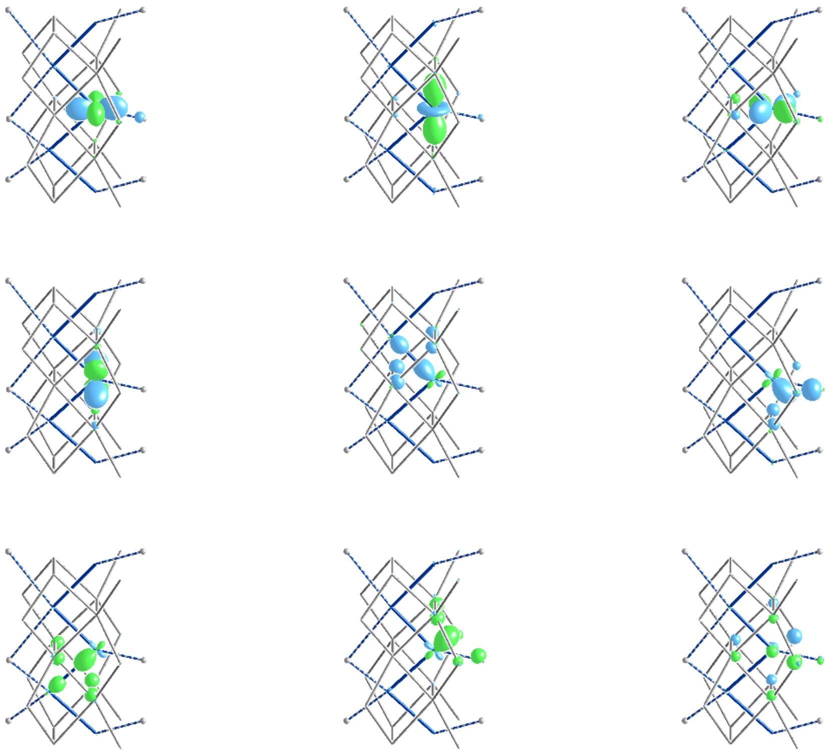

Figure S9 LC-raMO results for Fe3.

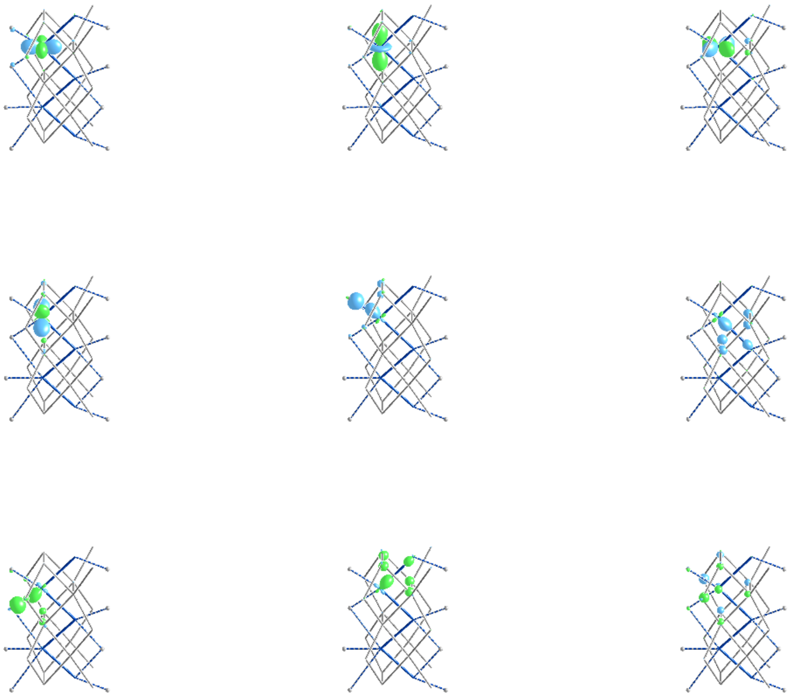

Figure S10 LC-raMO results for Fe5.

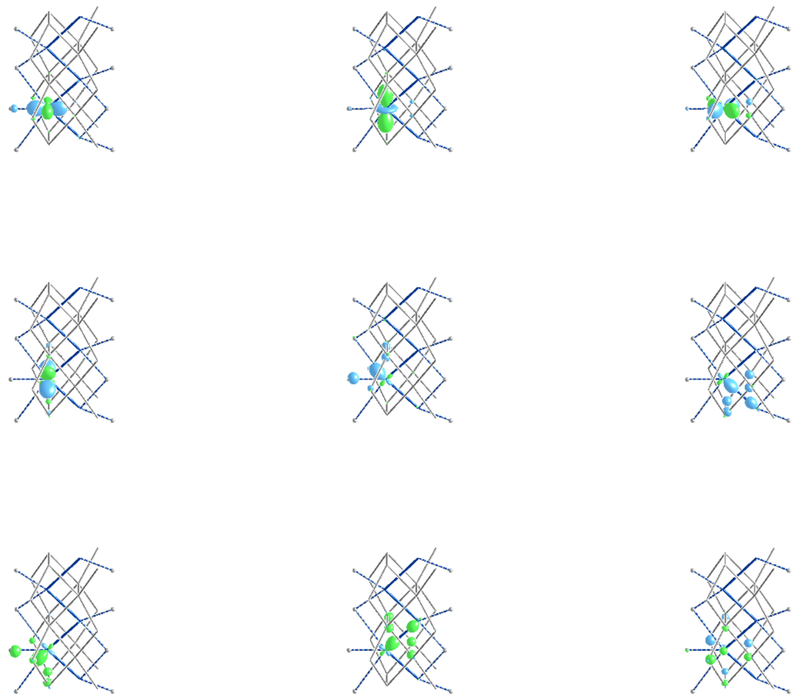

Figure S11 LC-raMO results for Fe6.

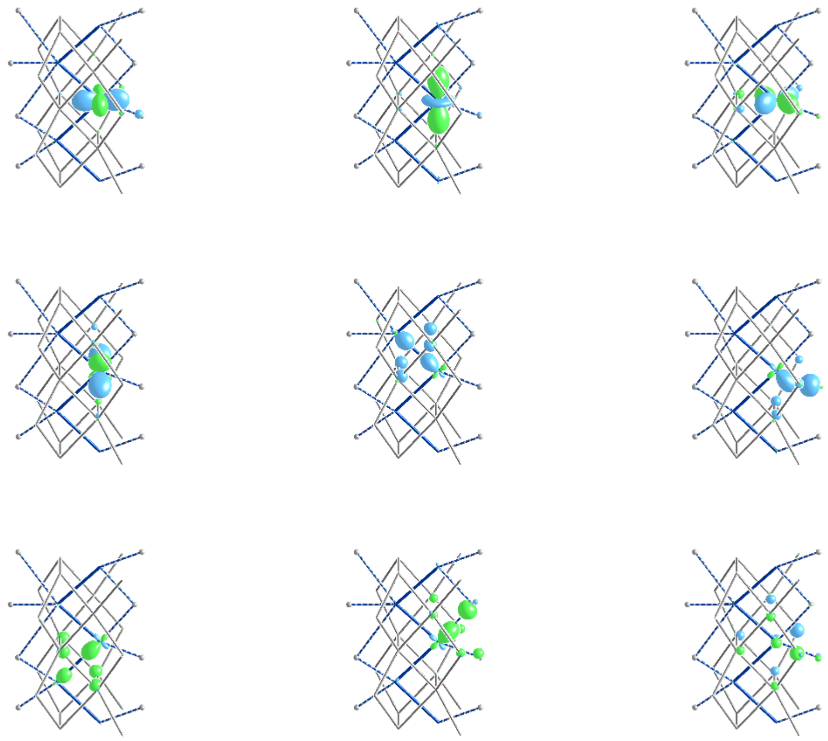

Figure S12 LC-raMO results for Fe7.

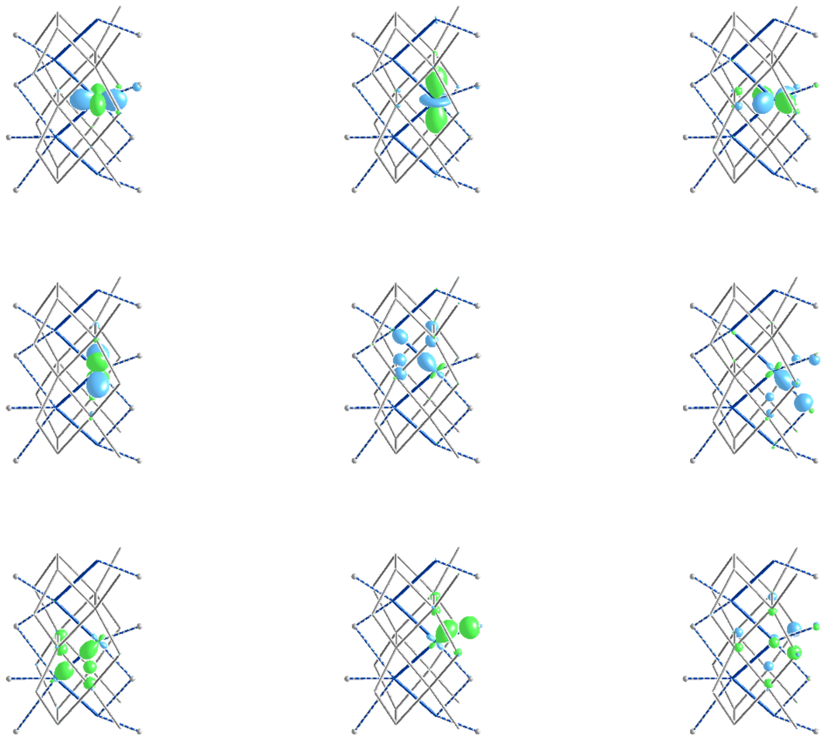

Figure S13 LC-raMO results for Fe8.

S9. raMO results for Fe Atoms in "Fe_2Al_5" (Model 2)

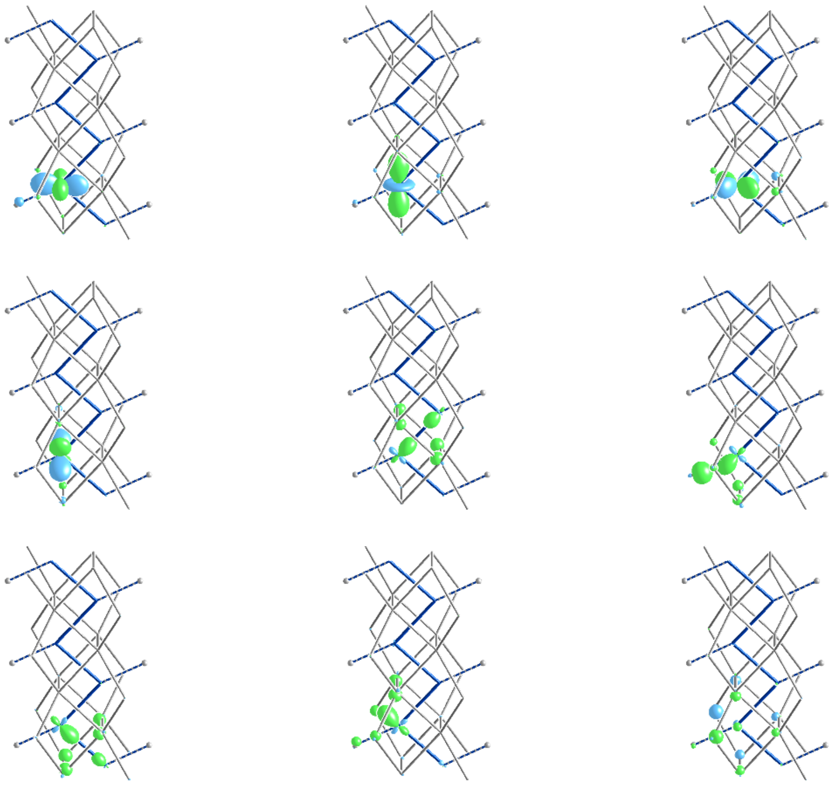

Figure S14 LC-raMO results for Fe1.

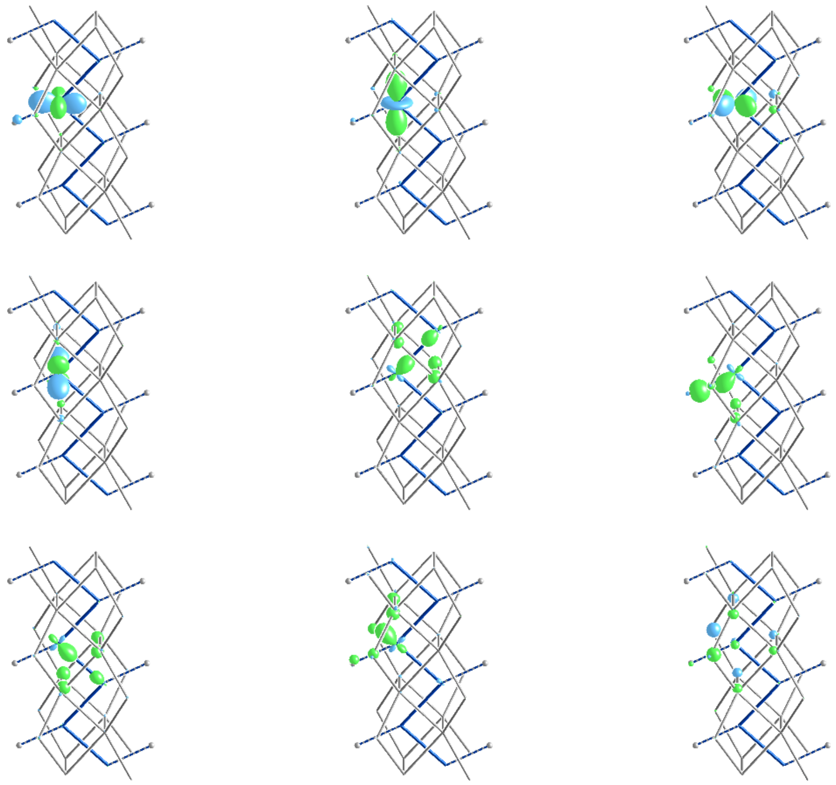

Figure S15 LC-raMO results for Fe2.

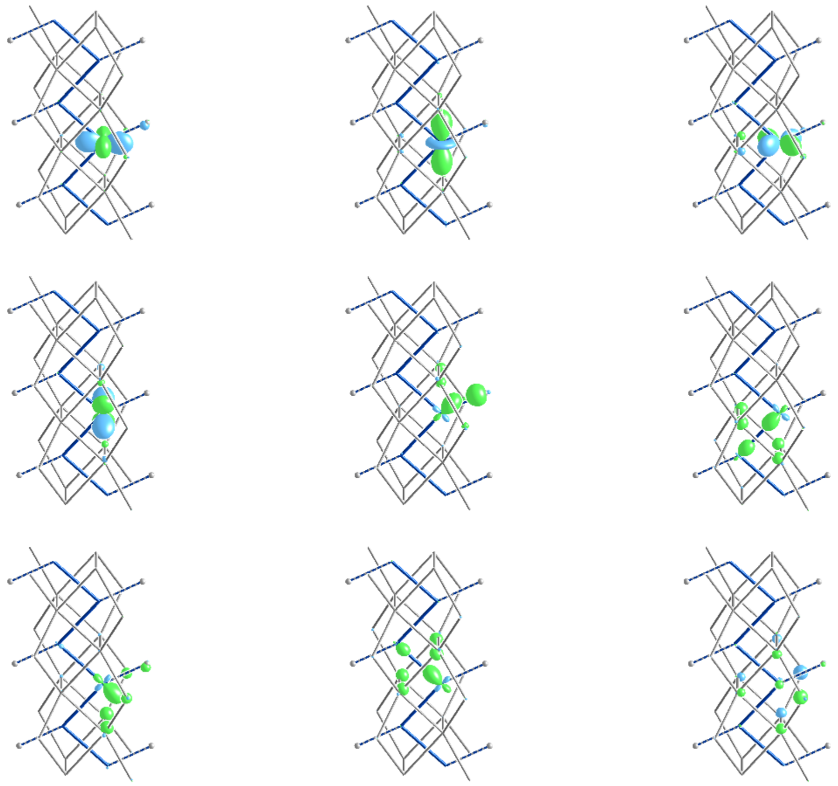

Figure S16 LC-raMO results for Fe3.

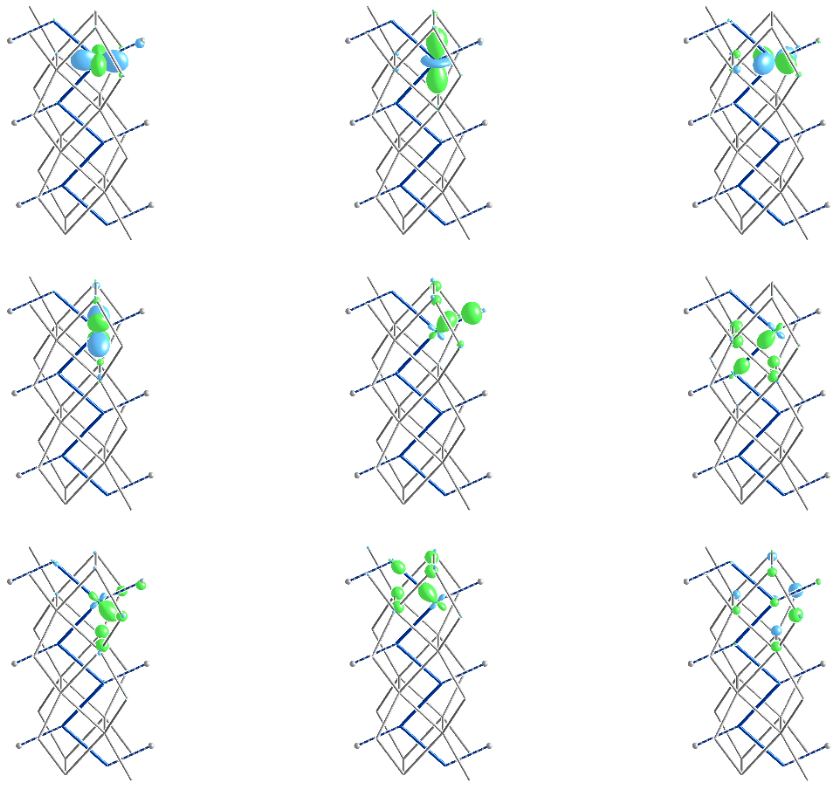

Figure S17 LC-raMO results for Fe4.

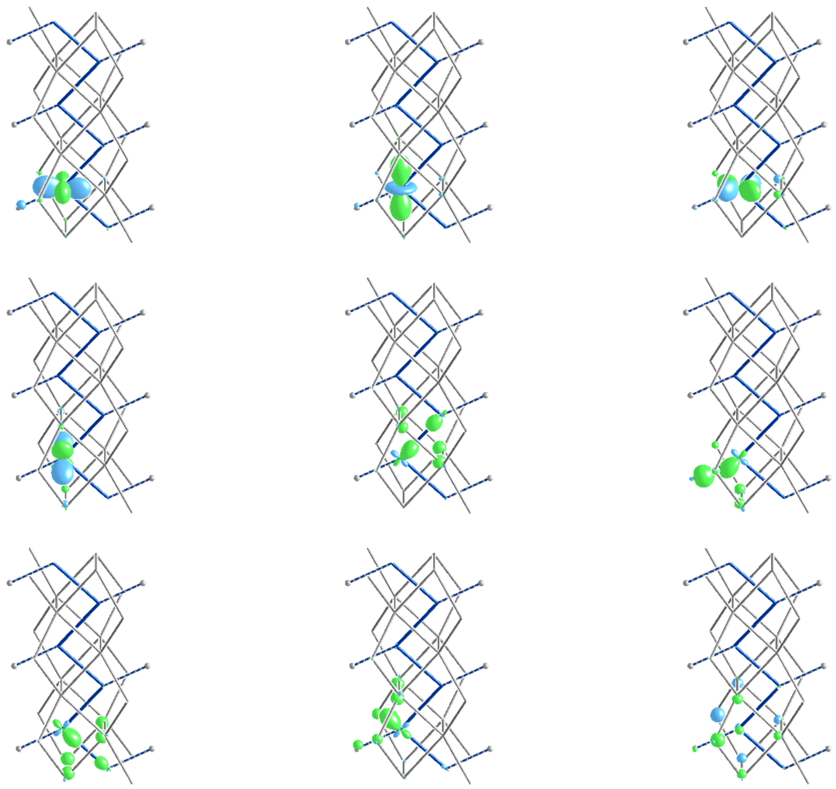

Figure S18 LC-raMO results for Fe5.

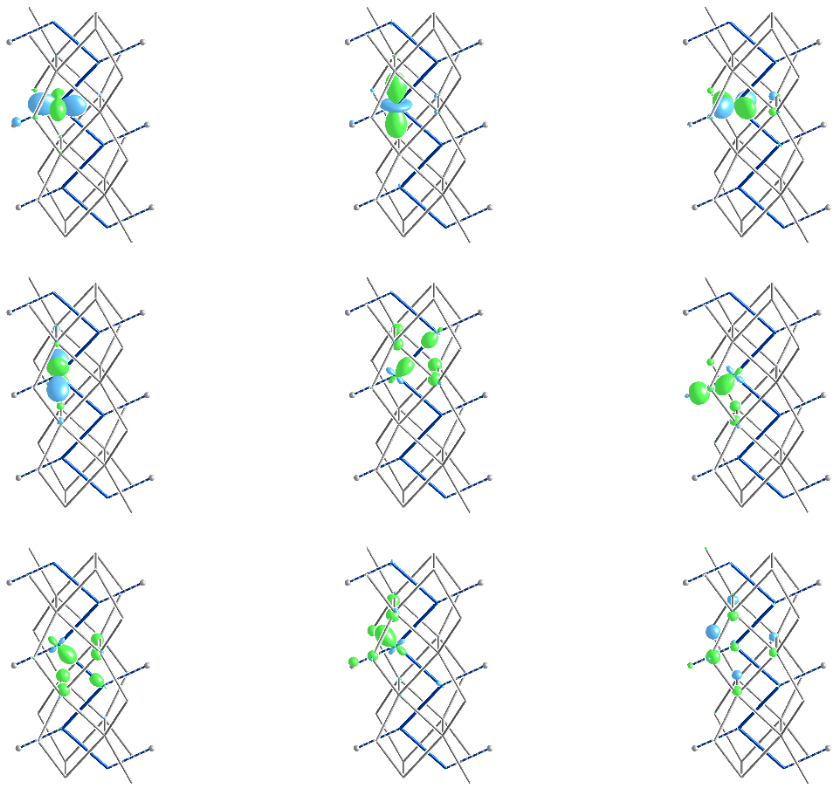

Figure S19 LC-raMO results for Fe6.

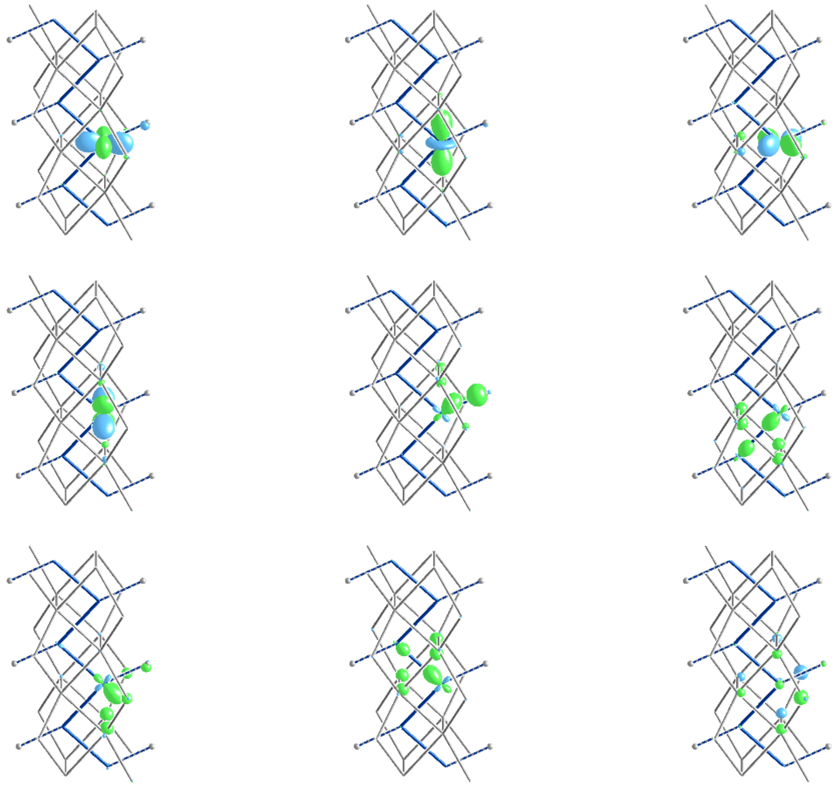

Figure S20 LC-raMO results for Fe7.

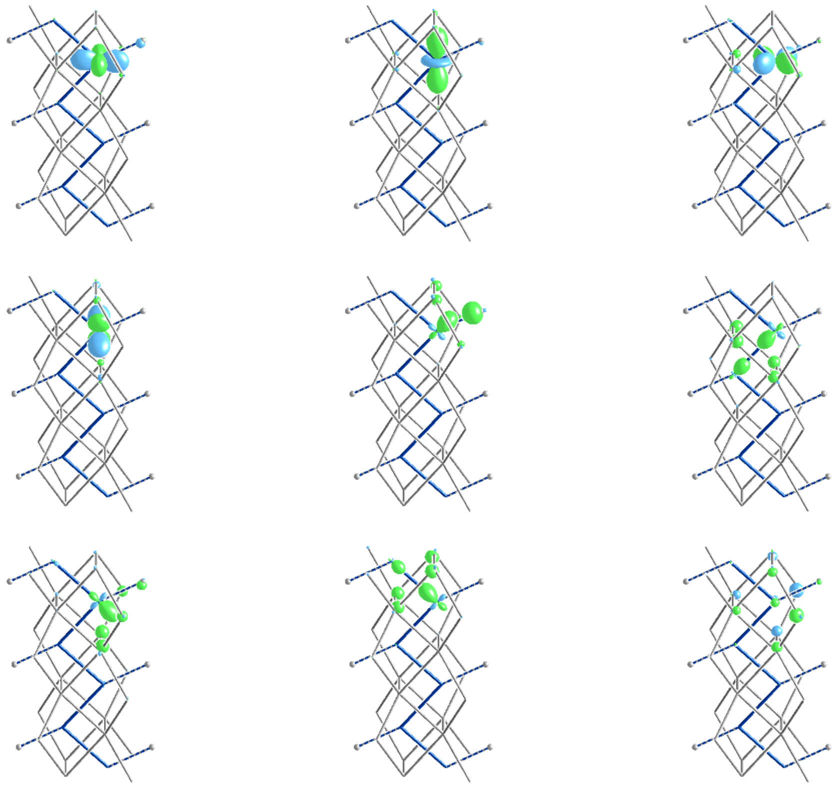

Figure S21 LC-raMO results for Fe8.

S10. Computational Details and Optimized Geometries for CP

Table S20 DFT-optimized (LDA, VASP) geometry for Model 1 ($Fe_2Al_{5.25}$).

Cell Vectors	*x* (Å)	*y* (Å)	*z* (Å)
**a** (Å)	7.39097	0.0	0.0
**b** (Å)	0.0	6.28425	0.0
**c** (Å)	0.0	0.0	8.24868
	x	*y*	*z*
Fe	0.0	0.672520	0.132732
Fe	0.0	0.672472	0.612749
Fe	0.0	0.331729	0.372541
Fe	0.0	0.325131	0.872675
Fe	0.5	0.166041	0.119235

Fe	0.5	0.166198	0.625951
Fe	0.5	0.846742	0.372850
Fe	0.5	0.821701	0.872493
Al	0.183219	0.349535	0.124257
Al	0.183079	0.348960	0.621019
Al	0.779208	0.620417	0.372769
Al	0.816193	0.656475	0.872574
Al	0.816781	0.349535	0.124257
Al	0.816921	0.348960	0.621019
Al	0.220792	0.620417	0.372769
Al	0.183807	0.656475	0.872574
Al	0.685767	0.856730	0.131864
Al	0.686134	0.857384	0.613415
Al	0.325803	0.173396	0.372521
Al	0.321767	0.157337	0.872658
Al	0.314233	0.856730	0.131864
Al	0.313866	0.857384	0.613415
Al	0.674197	0.173396	0.372521
Al	0.678233	0.157337	0.872658
Al	0.0	0.012081	0.034574
Al	0.5	0.518575	0.051650
Al	0.0	0.011592	0.711929
Al	0.0	0.950431	0.373002
Al	0.5	0.519416	0.691383

Table S21 DFT-optimized (LDA, ABINIT) geometry for Model 2 (Fe_2Al_5).

Cell Vectors	*x* (Å)	*y* (Å)	*z* (Å)

**a** (Å)	6.323891	0.0	0.0
**b** (Å)	0.000007	7.277169	0.0
**c** (Å)	0.179687	0.0	4.132048
	x	*y*	*z*
Fe	0.838550	0.000000	0.257931
Fe	0.161450	0.000000	0.742069
Fe	0.661450	0.500000	0.742069
Fe	0.338550	0.500000	0.257931
Al	0.655714	0.683823	0.245552
Al	0.500000	0.000000	0.500000
Al	0.344286	0.316177	0.754448
Al	0.655714	0.316177	0.245552
Al	0.344286	0.683823	0.754448
Al	0.844286	0.816177	0.754448
Al	0.155714	0.816177	0.245552
Al	0.844286	0.183823	0.754448
Al	0.155714	0.183823	0.245552
Al	0.000000	0.500000	0.500000

Table S22 DFT-optimized (LDA, ABINIT) geometry for Model 3 (Fe_2Al_5).

Cell Vectors	*x* (Å)	*y* (Å)	*z* (Å)
**a** (Å)	7.37629	0.0	0.0
**b** (Å)	0.0	6.38625	0.0
**c** (Å)	0.0	0.0	3.98734
	x	*y*	*z*
Fe	0.500000	0.682540	0.750000
Fe	0.500000	0.346671	0.250000
Fe	0.000000	0.846671	0.250000

Fe	0.000000	0.182540	0.750000
Al	0.500000	0.983725	0.250000
Al	0.000000	0.483725	0.250000
Al	0.314441	0.357110	0.750000
Al	0.685559	0.357110	0.750000
Al	0.185559	0.857110	0.750000
Al	0.814441	0.857110	0.750000
Al	0.185466	0.154322	0.250000
Al	0.814534	0.153422	0.250000
Al	0.314534	0.653422	0.250000
Al	0.685466	0.653422	0.250000

Table S23 DFT-optimized (LDA, VASP) geometry for Model 5 (Fe_2Al_5).

Cell Vectors	*x* (Å)	*y* (Å)	*z* (Å)
**a** (Å)	7.2956	0.0	0.0
**b** (Å)	0.0	6.3512	-0.0001
**c** (Å)	-0.0001	0.0	8.2225
	x	*y*	*z*
Fe	0.0	0.335333	0.119631
Fe	0.5	0.835333	0.130371
Fe	0.0	0.664667	0.380369
Fe	0.5	0.164666	0.369629
Fe	0.0	0.335333	0.619631
Fe	0.5	0.835333	0.630371
Fe	0.0	0.664667	0. 880369
Fe	0.5	0.164666	0.869629
Al	0.0	0.0	0.0
Al	0.5	0.5	0.25

Al	0.0	0.0	0.5
Al	0.0	0.0	0.75
Al	0.315742	0.846343	0.875992
Al	0.815750	0.346346	0.874007
Al	0.315742	0.153657	0.624008
Al	0.815750	0.653654	0.625993
Al	0.315742	0.846343	0.375992
Al	0.815750	0.346346	0.374007
Al	0.315742	0.153657	0.124008
Al	0.815750	0.653654	0.125993
Al	0.684258	0.153657	0.624008
Al	0.184251	0.653654	0.625993
Al	0.684258	0.846343	0.875992
Al	0.184251	0.346346	0.874007
Al	0.684258	0.153657	0.124008
Al	0.184251	0.653654	0.125993
Al	0.684258	0.846343	0.375992
Al	0.184251	0.346346	0.374007

Table S24 Computational parameters for ABINIT calculations for CP analysis.

Structure	*Energy cutoff*	*k-point vectors*[a]	*k-point shift*	*FFT grid*	*Total Energy*
Model 1 (Fe_2Al_5)	85.00 Ha	7 0 0<br>0 7 0<br>0 0 7	0.0 0.0 0.0	80 × 80 × 72	-52.006 Ha
Model 3 (Fe_2Al_5)	85.00 Ha	7 0 0<br>0 7 0<br>0 0 7	0.0 0.0 0.0	180 × 160 × 96	-103.986 Ha
Model 1 ($Fe_2Al_{5.25}$)	85.00 Ha	4 0 0<br>0 4 0<br>0 0 4	0.5 0.5 0.5	180 × 150 × 200	-210.154 Ha

[a] Three vectors that define a real-space super-lattice whose reciprocal lattice defines the k-point grid

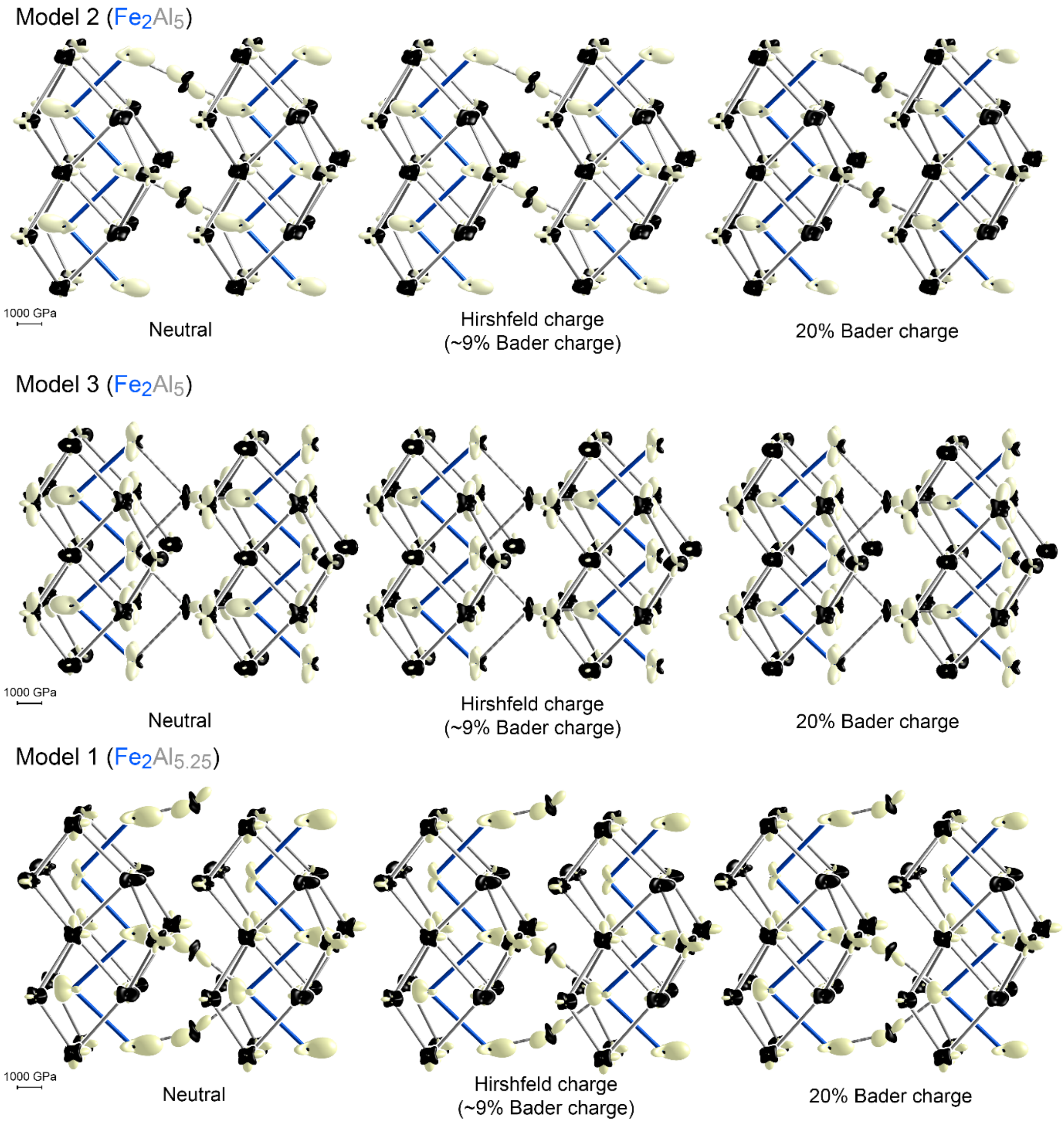

Figure S22 CP schemes of $Fe_2Al_{4+\delta}$ schemes with varying ionicities. Hirshfeld charges were determined using the Hirshfeld charge analysis carried out within the CP software package. All free ion electron densities were constructed using the Atomic Pseudopotentials Engine.

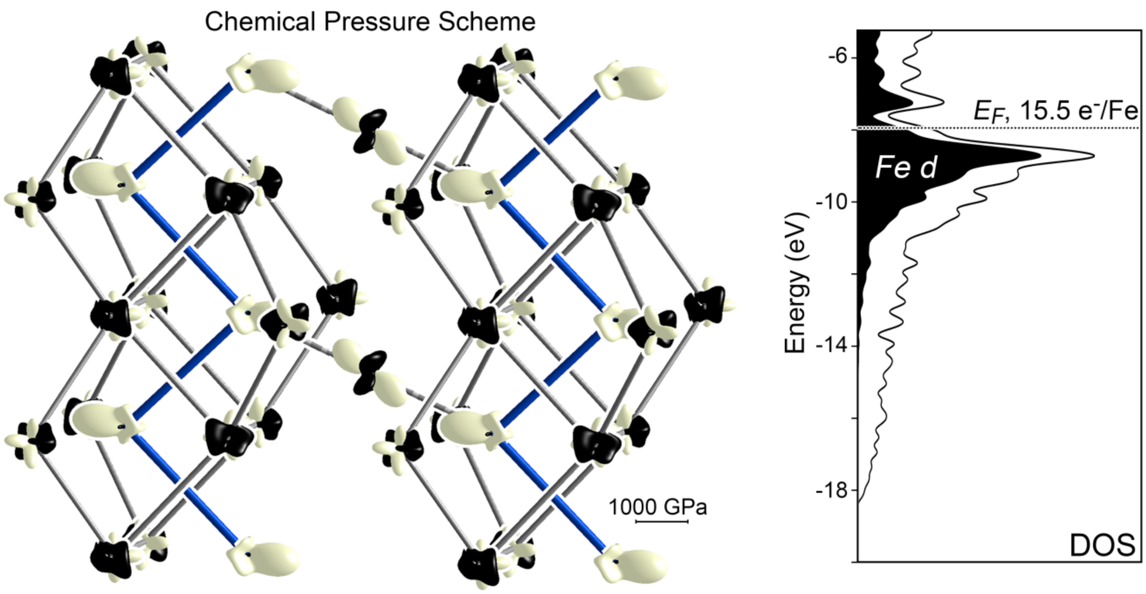

Figure S23 CP scheme (neutral ionicity) and electronic DOS distribution for Fe_2Al_5 Model 5 for comparison with the theoretical results for the other models.

Table S25 Computational parameters of response function calculations on Model 1 with ABINIT.

Structure	*Energy cutoff*	*k-point grid*	*k-point shift*	*FFT grid*	*q-points*
Model 1	85.00 Ha	3 × 3 × 3	0.0 0.0 0.0	80 × 80 × 72	8

References

Bruker (2016). *APEX3.* Bruker AXS Inc.,Madison, Wisconsin, USA.
Burkhardt, U., Grin, Y., Ellner, M. & Peters, K. (1994). *Acta Crystallogr. B* **50**, 313-316.
CrysAlisPro Software System, Version 171.37.35, 2015). Rigaku Oxford Diffraction: Yarnton, UK.
Goldbeck, O. K. v. (1982). *Iron-Binary Phase Diagrams*. Aachen: Springer-Verlag.
Grin, J., Burkhardt, U., Ellner, M. & Peters, K. (1994). *Z. Kristallogr. - Cryst. Mater.* **209**, 479.
Petříček, V., Dušek, M. & Palatinus, L. (2014). *Z. Kristallogr. - Cryst. Mater.* **229**, 345-352.
Sheldrick, G. M. (1996). *SADABS.* University of Göttingen,Germany.
Sheldrick, G. M. (2015a). *Acta Crystallogr.C* **71**, 3-8.
Sheldrick, G. M. (2015b). *Acta Crystallogr. A* **71**, 3-8.
